# Neurophysiological Treatment Effects of Mesdopetam, Pimavanserin and Amantadine in a Rodent Model of Levodopa‐Induced Dyskinesia

**DOI:** 10.1111/ejn.70032

**Published:** 2025-03-05

**Authors:** Abdolaziz Ronaghi, Tiberiu Loredan Stan, Sebastian A. Barrientos, Pär Halje, Azat Nasretdinov, Luciano Censoni, Sebastian Sulis Sato, Evgenya Malinina, Joakim Tedroff, Nicholas Waters, Per Petersson

**Affiliations:** ^1^ Department of Medical Translational Biology Umeå University Umeå Sweden; ^2^ The Group for Integrative Neurophysiology and Neurotechnology, Department of Experimental Medical Science Lund University Lund Sweden; ^3^ Integrative Research Laboratories Sweden AB Göteborg Sweden

**Keywords:** LFP, multi‐electrode, oscillations

## Abstract

Levodopa provides effective symptomatic treatment for Parkinson's disease. However, nonmotor symptoms are often insufficiently relieved, and its long‐term use is complicated by motor fluctuations and dyskinesia. To clarify mechanisms of levodopa‐induced dyskinesia and pharmacological interventions aimed at reducing dyskinetic symptoms, we have here characterized the neurophysiological activity patterns in sensorimotor and cognitive‐limbic circuits in dyskinetic rats, comparing the effects of amantadine, pimavanserin, and the novel prospective antidyskinetic and antipsychotic treatment mesdopetam. Parallel recordings of local field potentials from 11 cortical and subcortical regions revealed suppression of narrowband gamma oscillations (NBGs) in sensorimotor structures by amantadine and mesdopetam in conjunction with alleviation of dyskinetic signs. Concomitant gamma oscillations in cognitive‐limbic circuits were not directly linked to dyskinesia and were not affected by antidyskinetic treatments to the same extent, although treatment‐induced reductions in functional coupling were observed in both sensorimotor and cognitive‐limbic circuits, in parallel. In a broad frequency spectrum (1–200 Hz), mesdopetam treatment displayed greater similarities to pimavanserin than to amantadine. These findings point to the reduction of NBGs as a valuable biomarker for the characterization of antidyskinetic treatment effects and provide systems‐level mechanistic insights into the antidyskinetic efficacy of mesdopetam, with potential additional benefits for the treatment of Parkinson's‐related psychosis.

## Graphical abstract

By employing large‐scale recordings of brain activity in a rat model of Parkinson's disease, Abdolaziz et al. characterize brain activity patterns associated with levodopa‐induced dyskinesia. The study reveals distinct LFP oscillation patterns in the high gamma band in sensorimotor and cognitive‐limbic circuits arising from pharmacotherapy that are linked to antidyskinetic treatment effects.

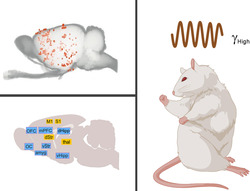

**Abbreviations**
AIMs: abnormal involuntary movements
amyg: amygdala
dHipp and vHipp: dorsal and ventral hippocampus
dStr and vStr: dorsal and ventral striatum
GLME: generalized linear mixed effects
i.p.: intraperitoneally
LID: levodopa‐induced dyskinesia
mPFC: medial prefrontal cortex
NBGs: narrowband gamma oscillations
OC: olfactory cortex
OFC: orbitofrontal cortex
PD: Parkinson's disease
M1: primary motor cortex
S1: primary somatosensory cortex
s.c.: subcutaneously
thal: thalamus

## 1 Introduction

Parkinson's disease (PD) is characterized by a severe loss of dopaminergic innervation to cortico‐basal ganglia circuits, and the dopamine precursor levodopa is the most widely used symptomatic treatment for PD. Unfortunately, long‐term levodopa treatment often leads to the development of motor fluctuations and dyskinesia that severely impacts the experienced quality of life (Perez‐Lloret et al. 2017). For these reasons, strategies to reduce the side effects of levodopa treatment, while maintaining the antiparkinsonian therapeutic effect, are of significant clinical importance. To date, amantadine (Blanpied et al. 2005; Shen et al. 2020) is the only approved drug for the treatment of dyskinesia. Amantadine was first discovered as an antiviral agent for influenza A, later in the 1960s it was discovered to also have antiparkinsonian effects (Schwab et al. 1969). Amantadine has a rich pharmacology interacting with a number of neuroreceptors, transporters, and ion channels (Danysz et al. 2021). The mechanism of action of amantadine in PD is not fully understood but likely includes interactions at several targets. Effects exerted by amantadine that may be associated with its antiparkinsonian and antidyskinetic properties are increased dopamine release and inhibition of NMDA receptors (Nikolaus et al. 2019). While many patients experience reduced problems with levodopa‐induced dyskinesia (LID) when co‐treated with amantadine (Pahwa et al. 2017), its wider use is limited by side effects including neuropsychiatric symptoms such as visual and auditory hallucinations and anxiety (Schwab et al. 1969; Pahwa et al. 2015), and there have also been reports on tolerance development (Thomas et al. 2004). It is, however, important to underscore that independently of these drug‐induced side effects, several PD patients experience nonmotor complications, including, for example sleep, and mood disturbances and visual hallucinations as parts of their disease (Fénelon and Alves 2010; Isaacson and Citrome 2022). These problems are often exacerbated by dopamine replacement therapy (Fénelon and Alves 2010; Forsaa et al. 2010). Further complicating the situation, treatment options for psychosis are quite limited in PD due to tolerability concerns, including worsening of PD motor symptoms and/or increased risk of falls associated with the use of several antipsychotic drugs. Pimavanserin (a selective 5‐HT2A antagonist/inverse agonist), which has proven to be both safe and tolerable, is therefore often the preferred alternative for the treatment of Parkinson psychosis (Kianirad and Simuni 2017). The usefulness of selective 5‐HT2A receptor antagonism as a means of LIDs treatment has also been discussed (Hamadjida et al. 2017), but remains undetermined. Whereas some preclinical studies are partly supportive, others are not, and there is a lack of clinical data to back this indication. Thus, for example, the hitherto most selective 5‐HT2A antagonist EMD‐281,014 (> 2000‐fold vs. 5‐HT2C & other targets; Bartoszyk et al. 2003) failed to counter abnormal involuntary movements (AIMs) in L‐DOPA‐treated 6‐OHDA‐lesioned rats (Frouni et al. 2019) but did significantly reduce the severity of L‐DOPA dyskinesia in (MPTP)‐lesioned marmosets (Kwan et al. 2019)—although only to a similar degree of about 60% across different doses tried (0.1–10 mg/kg). Pimavanserin does not worsen motor functioning in patients with Parkinson's‐related psychosis, and a randomized study on its possible effects on dyskinesia in PD has been carried out (NCT00086294; completed late 2007), but the reporting of results is still pending. This notwithstanding, it appears highly relevant to further explore potential treatments combining antidyskinetic as well as antipsychotic effects, given that PD patients often experience both dyskinesia and hallucinations.

In this perspective, targeting the dopamine D3 receptor (D3R) may represent an interesting alternative. Targeting the D3R has been shown to dampen both the acute expression of LIDs and prevent their development in both rodents (Lanza and Bishop 2021; Kuo et al. 2024; Wang and Zhang 2024) and nonhuman primates (Bézard et al. 2003; Visanji et al. 2009; Oh et al. 2022), and dampening striatal D3R expression results in significantly attenuated development of LIDs (Solís et al. 2017; Lanza and Bishop 2021). On a mechanistic level, the beneficial effects of targeting the D3R for LIDs have been suggested to be derived from inhibiting functional D1R‐D3R heteromeres (Fiorentini et al. 2008; Solís et al. 2017) that have been shown to be significantly increased as a consequence of chronic L‐DOPA treatment (Quik et al. 2000; Farré et al. 2015; Lanza and Bishop 2021), and to correlate to severity of LIDs (Scheggi et al. 2020). Heteromerization is also believed to result in potentiation of D1R‐mediated signaling (Fiorentini et al. 2008; Farré et al. 2015), which has been demonstrated as being central in the development of LIDs (Cenci et al. 1998; Picconi et al. 2003; Aubert et al. 2005; Pavón et al. 2006; Darmopil et al. 2009).

Mesdopetam is a dopamine receptor antagonist with a preference for D3Rs and physicochemical agonist‐like binding properties. Classical D3R antagonists are large and lipophilic chemical structures (Maramai et al. 2016), while mesdopetam more mimics D3R agonist structures and thereby most likely interfere with the D3Rs with a different binding mode. Indeed, mesdopetam has shown evidence of both antidyskinetic and antipsychotic‐like properties in animal models of PD and antidyskinetic effects in PD patients (Antonini et al. 2025; Svenningsson et al. 2018; Waters et al. 2020; Stan et al. 2024; Wang and Zhang 2024).

Thus, understanding the treatment mechanisms of amantadine, pimavanserin, and mesdopetam could be an important first step towards therapies aimed at reducing the spectrum of side effects of dopamine replacement therapy. However, characterizing the effects on brain activity patterns of different drugs in the widely distributed neuronal circuits that are known to contribute to LID and other side effects is experimentally extremely challenging. Fortunately, the recent development of advanced methods for neurophysiological recording in brain networks has now made such studies possible—providing a new powerful tool to evaluate pharmacological treatment effects in the brain of awake behaving subjects (Ivica et al. 2014; Tamte et al. 2016). We have here adapted these techniques to obtain large‐scale recordings in freely moving rats during LID with the aim of characterizing the potential antidyskinetic treatment effects of amantadine, pimavanserin, and mesdopetam with respect to the neurophysiological activity changes induced, together with potential concomitant reductions of dyskinesia, expressed as AIMs and hyperkinesia.

## 2 Materials and Methods

### 2.1 Animals and Housing

Adult female Sprague–Dawley rats (250–350 g, approximately 4‐months old) were used for the experiments. The rats were sourced from Taconic Biosciences, Denmark for the Lund experiments, and from an in‐house colony for Umeå experiments. Animals were housed in open plastic cages at Umeå Center for Comparative Biology's (*n* = 4) and at Medicon Village, Lund University (*n* = 4). Animals were kept in an ambient temperature at 22°C–24°C, on a 12‐h light/dark cycle, with food and water available *ad libitum*. All procedures adhered to national and local animal welfare guidelines and were approved by the Swedish Ethical Committee for Northern Sweden (Dnr. A15‐2018) and the Malmö/Lund ethical committee in advance (Ethic permit number 9/1801689/2019).

### 2.2 6‐OHDA Lesions and Procedures for Surgical Implantations

For 6‐OHDA lesions, the animals were anesthetized with fentanyl (0.2 mg/kg) and medetomidine (Dormitor, 0.1 mg/kg), which were administered intraperitoneally (i.p.) or subcutaneously (s.c.). Two stereotaxic injections of 6‐hydroxydopamine (6‐OHDA) hydrochloride (Sigma, 6.0 μg/μl in 0.02% ascorbate saline) were given into the medial forebrain bundle of the right hemisphere. The first injection of 2.5 μL was delivered at a rate of 1 μL/min at the coordinates: AP: −4.0, ML: −1.2, and DV: −7.8, with the tooth bar set at −4.5. The second injection of 2 μL was administered at the same rate at the coordinates: AP: −4.0, ML: −0.8, and DV: −8.0, with the tooth bar set at +3.4. Carprofen (5 mg/kg, s.c.) was administered to relieve perioperative and postoperative pain and to attenuate the inflammatory response, continuing until postoperative Day 5. Other postoperative care included daily monitoring of body weight and soft food supplements.

For electrode implantations, anesthetic/analgesic procedures and postoperative care were similar to those used for 6‐OHDA lesions. In addition, to minimize infection risk, enrofloxacin (5 mg/kg, s.c.) was administered 2 h before surgery and treatment continued until postoperative Day 7. During surgery, microwire electrodes were implanted in both hemispheres using stereotaxic procedures. The implant was secured with dental acrylic attached to anchoring screws (Angtho's AB, Lidingö, Sweden, Ø 2 mm × 3 mm each) inserted into the skull. A 200‐μm thick silver wire was attached to the posterior skull screws, covered with conductive silver paint, and used as a ground connection from the animal to the recording system. For the first three postoperative hours, animals recovered on a heating blanket. Postoperative care was provided for 5 days as described for 6‐OHDA lesions. The animals were allowed to recover for at least 2 weeks after surgery before initiation of the experimental protocol.

### 2.3 Recording Electrodes

Recording arrays were built according to the procedure previously described by Ivica et al. (Ivica et al. 2014). Formvar‐insulated tungsten wires (33‐μm diameter, California Fine Wire Co., CA) were arranged into 30 groups of arrays (15 groups per hemisphere targeting different brain areas with 2–8 wires per target) with 250‐μm wire spacing in each horizontal dimension and a wire length corresponding to the depth of the recording target. After verification of tip locations with CT (Censoni et al. 2022), wires in the two hemispheres were grouped according to functional relatedness into 27 main structures, of which 15 were deemed to have a sufficient number of electrode pairs (Figure S1). Four of these structures (claustrum, insula, ventral pallidum, and parietal cortex) were, however, not included in the further analyses because they were not sufficiently covered across animals (criterion used: recordings from the lesioned hemisphere in at least 3 animals; Figure S1). Thus, the remaining 11 regions used throughout the study were (with abbreviations used in parentheses) amygdala (amyg), dorsal and ventral hippocampus (dHipp and vHipp), dorsal and ventral striatum (dStr and vStr), olfactory cortex (OC), orbitofrontal cortex (OFC), medial prefrontal cortex (mPFC), primary motor cortex (M1), primary somatosensory cortex (S1), and thalamus (thal).

### 2.4 L‐DOPA Priming Procedure

**FIGURE 1:**
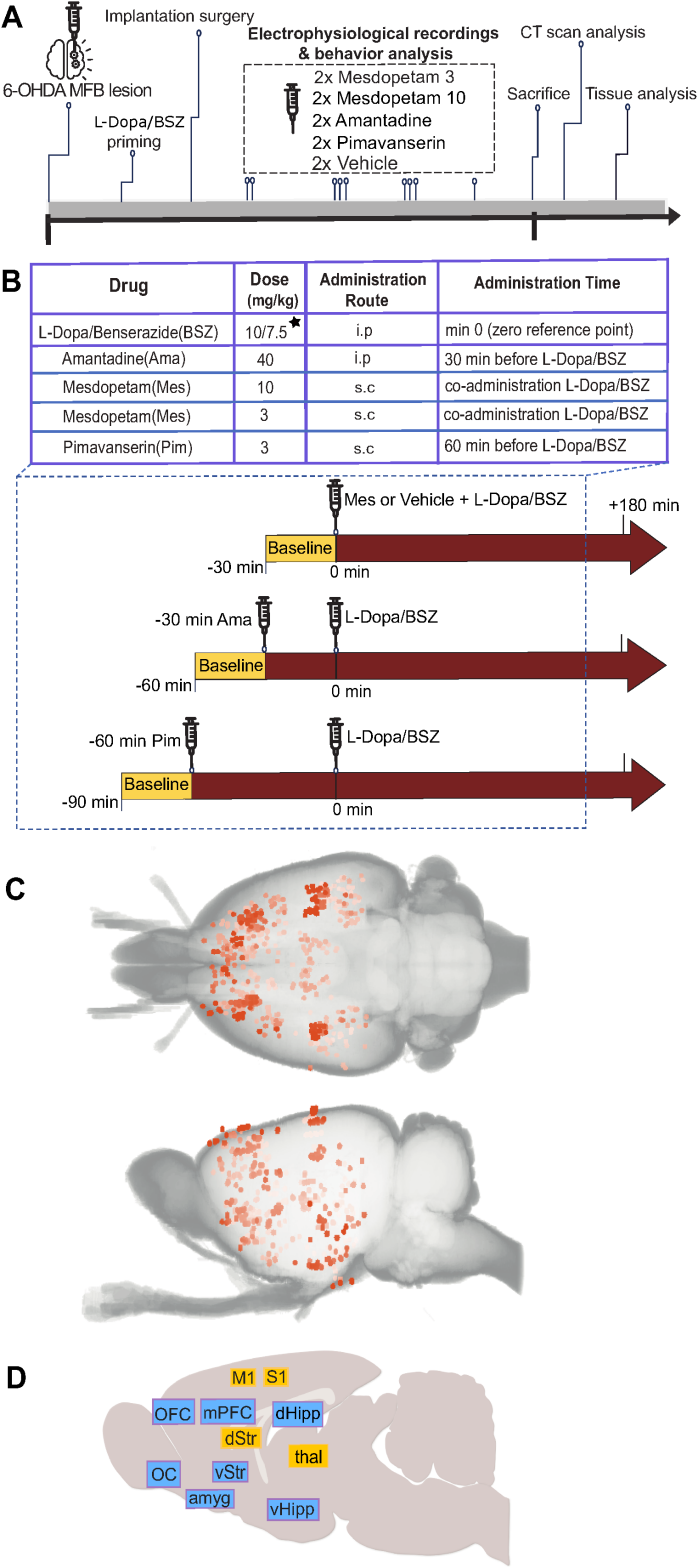
Overview of experiment design and brain structures recorded. (A) Timeline indicating the order of experimental interventions. Period from lesion to sacrifice corresponds to approximately 3 months. (B) Doses, administration routes, and relative injection times of the compounds investigated. [*] in two rats 20‐mg/kg L‐DOPA was used. (C) Top and side view of recording locations (*n* = 619 in total), reconstructed from postmortem CT scans (overlapping sites are indicated by higher color saturation). (D) Overview of the main 11 structures analyzed. Blue and yellow colors denote structures with predominantly cognitive/limbic and sensorimotor‐related functions, respectively.

When fully recovered after lesion the procedure, animals were treated with daily doses of levodopa (L‐DOPA)/benserazide (Sigma‐Aldrich, Sweden), dissolved in saline and administered at a dose of 10/7.5 mg/kg i.p. (Schintu et al. 2020), for 14 days (Figure 1A). Under these sessions, signs of hyperkinesia/dyskinesia were monitored, and animals that showed moderate to high levels of dyskinetic symptoms were implanted with an electrode array.

### 2.5 Pharmacological Experiments

At least 2 weeks after the electrode array implantation, animals were subjected to experimental pharmacological treatments during electrophysiological and behavioral recordings (Figure 1B). Vehicle experiments (saline and saline + DMSO) were conducted first and last in each series of experiments, whereas the other treatments were performed in a pseudorandomized order. The experiments were performed in each rat at least 2 days apart to allow for drug washout. Drugs were administered at a volume of 1.0 mL/kg of the animal's body weight.

The NMDA receptor antagonist, amantadine hydrochloride (1‐aminoadamantane hydrochloride, AbMole Bioscience, Sweden), dissolved in 0.9% saline was administered (40 mg/kg i.p.) 30 min prior to L‐DOPA/benserazide (see, e.g., Bortolanza et al. 2016; Finlay et al. 2024). The selective 5‐HT2A antagonist/inverse agonist, pimavanserin (AbMole Bioscience, Sweden), was diluted first in DMSO to create a stock solution of 10 mg/mL, as follows: 10‐mg pimavanserin + 100‐uL DMSO + 900‐uL H_2_O. For the desired dose of 3 mg/kg, an aliquot from the stock solution was further diluted in saline and administered s.c. 60 min prior L‐DOPA/benserazide. The dose and time of administration for pimavanserin have previously been shown to reverse psychosis‐like behaviors in a rodent model of Parkinson's‐related psychosis without interfering with spontaneous motor behavior (McFarland et al. 2011; Hubbard et al. 2013). For mesdopetam (provided by IRLAB, Sweden), two different doses were evaluated, 3 and 10 mg/kg (Waters et al. 2020; Antonini et al. 2023). Mesdopetam was diluted in saline and administered s.c. and co‐administered with L‐DOPA, following procedures observed to yield significant antidyskinetic effects in previous studies (Waters et al. 2020).

### 2.6 Behavioral and Electrophysiological Recordings During Experimental Antidyskinetic Treatment

Behavioral and electrophysiological recordings were performed in a Faraday cage. Animals were placed in a transparent plastic cylinder (50–60 cm in diameter) on a glass surface. Digital videos from cameras positioned on the side as well as below or above the rat were synchronized to electrophysiological recordings using a pulse generator (Master‐8/9, AMPI). After a 10‐min acclimatization period in the cylinder, baseline recordings were obtained for 10–30 min. Subsequently, brain activity was recorded under the influence of different pharmacological agents. Depending on the experimental paradigm, different pharmacological compounds were administered (Figure 1B), and recording of electrical activity and locomotor activity continued up to 180 min.

### 2.7 Rating of AIMs

AIMs were assessed offline using an adapted version of previously described protocols (Angela Cenci and Lundblad 2007; Sebastianutto et al. 2016; Tamte et al. 2016). In brief, AIMs were classified into three subtypes: (a) orolingual dyskinesia: stereotyped jaw movements or contralateral tongue protrusion; (b) forelimb dyskinesia: repetitive jerky, dystonic, or grabbing movement of the contralateral forelimb; and (c) axial dyskinesia: twisted posture of the neck and upper body toward the side contralateral to the lesion. In general, for each subtype, both severity and amplitude scales were considered.

The AIM severity scale evaluates the proportion of observation time during which dyskinetic behaviors are present and includes the following subtypes: 0 = no dyskinesia; 1 = occasional signs of dyskinesia, which are present during less than half of the observation time; and 2 = frequent signs of dyskinesia that are present during more than half of the observation time. Dyskinetic movements displayed during the entire period were scored as 2.5. The AIMs amplitude scale is a complementary scale that further expands the dynamic range of the total dyskinesia score per session. The amplitude criteria considered for each subtype were the following: axial AIMs: (1) sustained deviation of the head and neck, at ∼30° angle; (2) sustained deviation of the head and neck, > 30 but ≤ 60° angle; (3) sustained twisting of the head, neck, and upper trunk > 60° but ≤ 90° angle; (4) sustained twisting of the head, neck, and trunk at maximal amplitude (angle > 90°), causing the rat to lose balance. Limb (forelimb) AIMs: (1) small movements of the paw around a fixed position; (2) movements resulting in a visible displacement of the whole limb either sideways or up‐and‐down; (3) large displacement of the whole limb with visible contraction of shoulder muscles; (4) vigorous limb displacement of maximum amplitude, with conspicuous contraction of both shoulder muscle groups and more distal extensor muscles. Orolingual AIMs: (1) twitching of facial muscles accompanied by small masticatory movements without opening the jaw; (2) twitching of facial muscles, accompanied by noticeable masticatory movements, occasionally leading to jaw opening; (3) movements with broad involvement of facial muscles and masticatory muscles; frequent jaw opening, occasional tongue protrusion; (4) all the above muscle categories involved to the maximal degree.

Subsequently, the products (severity × amplitude) of each dyskinesia subtype were summed to create a global AIMs score (axial/limb/orolingual; ALO) for each 1‐min observation period, occurring in 10‐min intervals, for a total of 180 min after L‐DOPA injection.

### 2.8 Automated Quantification of Spontaneous Motor Behavior

Video recordings were processed to extract coordinates for selected body parts using DeepLabCut (Mathis et al. 2018; Nath et al. 2019). In brief, separate networks were trained for the top and bottom camera using default settings and a minimum of 600 manually labeled frames each. The network was then applied to extract coordinates from all video recordings. Tracked body parts included the tail base, tail tip, and body center, as well as left and right forepaws, hind paws, and the mouth for the bottom camera network. For the top camera network, tracked parts included the left and right ears, top of the head, and nose.

To quantify the egocentric rotational behavior of rats, positional data detected using DLC were processed as follows. First, the x and y coordinates of key anatomical points on the head of the animal (nose/mouth for bottom camera and ears/head for top camera) and the center of rotation (tail base for bottom camera and body center for top camera) were separately averaged. To minimize noise, the coordinates of front and center positions were smoothed using a low‐pass filter with a 5‐Hz cutoff frequency. Subsequently, the vector angle of the front body part respect to the center was calculated for each video frame. Angles that changed continuously along a circumference subdivided into eight sectors were considered a complete rotation, and incomplete ones were discarded. Although both ipsi‐ and contralateral rotations (with respect to the lesion side) were detected, only the latter were considered for further analysis. Contralateral rotation events were counted starting from the time of L‐DOPA administration and summed over a 10‐min sliding window without overlap. To compare the effect of the treatments on inhibiting L‐DOPA‐induced contralateral rotations, the rotational events (expressed as counts per minute) were averaged during two specific periods: the peak effect period (40–80 min postadministration) and the late period (120–160 min postadministration).

### 2.9 Neurophysiological Signal Acquisition and Processing

Electrical signals were filtered to obtain signals between 1 and 7500 Hz, sampled and digitized at 30 kHz by the amplifier in the headstage, and recorded by the Intan RHS2000 and RHD2000 systems. Signals were subsequently downsampled to 2000 Hz, off‐line.

Bipolar local field potential (LFP) time series were computed from separate pairs of electrodes located in the same structure. Notably, by using this method, we made sure that external sources contributed minimally to the local voltage fluctuations via volume conductance, thus better separating out the local sources. Time‐frequency power spectral densities (i.e. spectrograms) were calculated over the 0–300 Hz frequency range with 50% overlapping 8‐s Hanning windows (0.5‐Hz resolution) for each of the time series. To emphasize oscillatory components in the power spectrum, we used irregular resampling (Wen and Liu 2016). In brief, this method is based on the principle that the power spectrum of arrhythmic components is unaffected by time series resampling, thus enabling the separation of the fractal component of the spectrum by resampling the time series multiple times. Hence, by normalizing the total spectrum to the nonperiodic fractal component, it is possible to construct a power spectrum measure that emphasizes truly rhythmic activity.

### 2.10 Peak Detection

To detect high‐frequency oscillations (HFOs) and gamma bands oscillations, we defined a parametric model,

$$y\left(f\right)=A^{*}\exp\left(-\left(\left(f-B\right)/C\right)2\right)+\mathrm{Df}+E.$$

which was fitted (Matlab *fit* function) to the spectra obtained by averaging together the spectra of individual electrode pairs from the same structure. The parameters *A* (peak height), *B* (peak frequency), *C* (peak width), *D* (inclination of flat background), and *E* (offset of flat background) were estimated such that the model y(f) fitted the spectrum optimally in the least‐squares sense. This allowed us to detect HFO/gamma peaks automatically by defining thresholds for the goodness‐of‐fit (*R* ^2^) and the fitted parameters. Typical conditions for a positive detection were *R* ^2^ > 0.2, 2 < A < 100 dB, and 115 < B < 170 Hz (HFO) or 30 < B < 70 (gamma), 1 < C < 20 Hz, −1 < D < 1, and −10 < E < 10. In the event of positive detections, the model was also used to quantify peak frequency parametrically.

Band power was calculated as the mean power over a 20‐Hz band centered on the peak frequency. The peak frequency was defined individually for each recording session and each structure as the median of the *B* parameter across the recording session.

### 2.11 Description of Phase Analysis

To quantify the instantaneous phase of NBGs, monopolar LFP time series were bandpass filtered ±5 Hz around the median NBG frequency of each recording (as determined by the *B* parameter above). We used a 64‐order FIR filter (Matlab *fir1* function) backwards and forwards to ensure zero phase lag (Matlab *filtfilt* function). The bandpassed signal was Hilbert transformed into the complex‐valued analytical signal *z(t)* (Matlab *hilbert* function) and instantaneous phase could then be calculated as $\varphi \left(t\right)=\text{argz}\left(t\right)$.

The phase relation between two wires *i* and *j* was calculated as $\left(\mu_{\mathrm{ij}},\kappa_{\mathrm{ij}}\right)=f\left(\varphi_{i}\left(t\right)-\varphi_{j}\left(t\right)\right)$, where *f* is a function that estimates the von Mises distribution (*circ_vmpar*, Matlab CircStat toolbox; Berens 2009). To estimate how consistent the phase difference was between two structures (a metric of functional connectivity), the median of the concentration parameter κ was calculated for all wire pairs belonging to the same two structures.

### 2.12 Brain State Comparisons Based on Spectral Correlations

For spectral correlation analysis, signals were collected from individual recording electrodes in 4‐s windows between 40–80 min and 120–160 min after L‐DOPA administration. Spectra in the range 1–200 Hz s were then normalized to the aperiodic part of the power density spectrum and were averaged for all recording sites in the same structure. For each mesdopetam experiment, pairwise Pearson correlations were calculated, for each structure, between the mesdopetam spectrum and the average spectrum for the amantadine and pimavanserin recordings, respectively.

### 2.13 X‐Ray Tomography to Verify Electrode Positions

To verify the accurate anatomical targeting of the studied brain structures, we utilized a newly developed semiautomated method, employing acquired Computer Tomography (CT) images (Censoni et al. 2022). After the completion of recording experiments, the rats were anesthetized with a lethal dose of sodium pentobarbital (100 mg/kg i.p., Apoteksbolaget AB, Sweden). Following transcardial perfusion with 0.9% saline and subsequent fixation with 4% paraformaldehyde, the animals were decapitated *postmortem*. The heads, along with the preserved electrode implants, were then immersed in 4% paraformaldehyde for 24 h before being transferred to a 25% sucrose solution to prevent excessive tissue dryness and shrinkage. CT scans were conducted using the MILabs XUHR system (MILabs, the Netherlands) and Mediso Nanoscan PETCT scanner (Mediso, Hungary), with the heads positioned such that the wires were perpendicular to the photon beam (Censoni et al. 2022). The scanned volumes were registered to an anatomical atlas using bone landmarks, and the image coordinates of the electrode tips were converted to stereotaxic atlas coordinates. Finally, the wire tips were assigned appropriate anatomical labels based on their location in the atlas (Paxinos and Watson 2007).

### 2.14 Statistical Analyses

**FIGURE 2:**
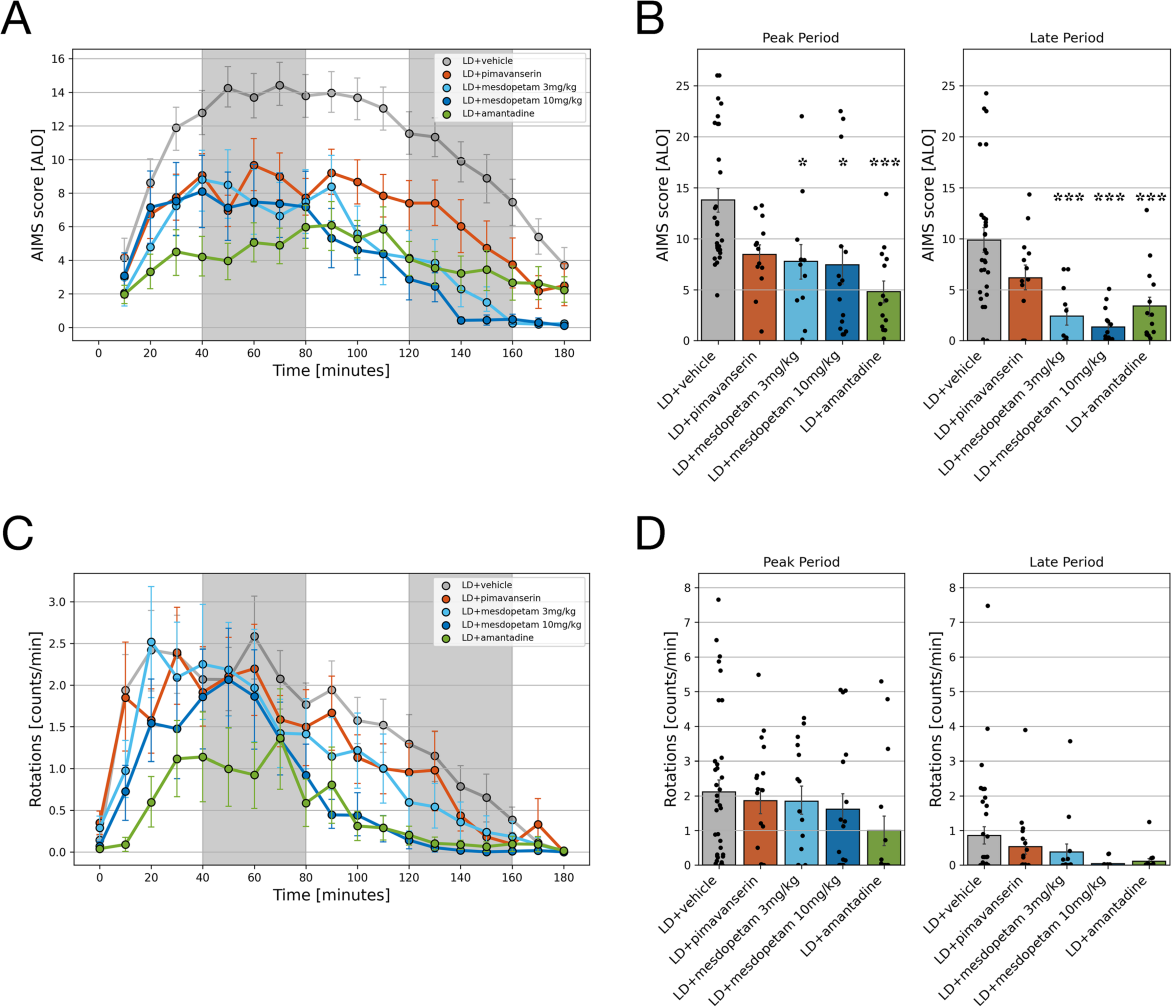
Quantification of abnormal involuntary movements and hyperkinesia. (A) Average global scores including axial, limb, and orolingual (ALO) AIMs sampled every 10 min after L‐DOPA injection (time = 0 min) under each drug treatment. (B) Average global AIMs score during the peak and late period (left and right panel, respectively) after L‐DOPA injection. (C) Average of the total contralateral rotations counted over consecutive 10‐min periods upon each treatment administration. (D) Average of contralateral rotations per minute during the peak and late period (left and right panel, respectively) after L‐DOPA injection. Filled circles indicate individual recording sessions in each experimental condition (for AIMs, [*n* = 29, 13, 11, 15, and 14] and for rotations [*n* = 36, 18, 15, 17, and 16]). Error bars denote SEM, and significance in Panels (B) and (D) are marked by [*] a significant difference to L‐DOPA + vehicle (GLME, Tukey‐HSD posttest). Significance in Panel (B) is as follows: *p < 0.05 and ***p < 0.001.

Statistical analysis on either AIMS or Rotational behavior was performed using a generalized linear mixed effects (GLME) model in Python 3.10. A GLME was fitted for each experimental period with the formula: *Behavior ~ Drug + (1 + SessionOrder|Animal)*. In this design, *Drug* was the primary fixed factor, while *SessionOrder* and *Animal* were included as random factors, with each rat tested twice under each drug condition. The term *SessionOrder* refers to the sequence in which the experimental conditions were applied to each animal, accounting for potential effects due to the order of testing. The model evaluates the impact of different drug treatments on the response variable (e.g., Dyskinesia Score or Rotations, as shown in Figure 2), using LD + vehicle as the baseline condition. The Drug effect quantifies how each treatment differs from the LD + vehicle reference level. Including Animal as a random factor accounts for individual variability, allowing each rat to have its own baseline response. This accommodates any intrinsic differences between the subjects such as baseline activity levels or electrode placement. Regarding repeated measures, since each rat was tested twice under each drug condition, the model treats these repeated observations as dependent within each animal. Post‐hoc pairwise comparisons between drug treatments were conducted, with *p*‐values adjusted for multiple comparisons using the Tukey‐HSD test. Single sessions were considered as replicates under this design. Sample sizes are described in the main text and in the respective figure legends. Significant *p*‐values are represented with asterisks in the order *p* < 0.05 *, *p* < 0.01 **, and *p* < 0.001 ***. *p*‐values over 0.05 were considered nonsignificant. Error bars represent mean ± SEM. Other statistical tests used are described in relation to the presentation of the respective results.

## 3 Results

### 3.1 Experimental Design and Anatomical Mapping of Recorded Sites

In total, eight female Sprague–Dawley rats were exposed to unilateral 6‐OHDA medial forebrain bundle lesions, following established protocols known to cause marked dopaminergic denervation throughout forebrain structures, including the ipsilateral cerebral cortex and dorsal striatum (Halje et al. 2012; lesions were also verified behaviorally as indicated by the display of a significant ipsilateral turning bias in spontaneous motor behavior, Figure S2). Following 2 weeks of daily levodopa injections (10 mg/kg), it was confirmed that all eight rats had developed moderate to severe LID (as assessed by standardized rating scales; Cenci and Lundblad 2007), and animals were chronically implanted with electrode arrays for recording of brain activity (Figure 1A). In the subsequent pharmacological experiments, L‐DOPA doses were adjusted on an individual basis to ensure dyskinesia of comparable severity in all subjects for the full recording period (10/7.5 mg/kg in all except two rats that instead received 20/7.5 mg/kg, L‐dopa/benserazide). In different recording sessions, L‐DOPA was combined with either vehicle, amantadine (40 mg/kg), pimavanserin (3 mg/kg), or mesdopetam (3 or 10 mg/kg), and behavior and brain activity patterns were recorded for 3 h, after a 20‐min baseline (Figure 1B; for simplicity, all three substances are here referred to as antidyskinetic compounds). To enable parallel recording of several sensorimotor and cognitive‐limbic structures potentially affected by the different treatments, specialized electrode arrays were developed that individually target 128 separate recording sites arranged in complex geometries (Ivica et al. 2014). At the end of the full experimental series, animal heads were scanned with CT, and detailed 3D reconstructions were created to identify the location of each electrode tip with high precision (Figure 1C; Censoni et al. 2022). These analyses showed that recording tips were distributed in 95 different brain structures across the eight rats (as defined by the Paxinos and Watson rat atlas of the brain; Paxinos and Watson 2007), divided approximately equally in the two hemispheres. Based on functional relatedness, these sites were subsequently merged into 27 groups, and for the further data analyses, 11 of these groups (covering sensorimotor and cognitive limbic structures) were selected, which all had enough electrodes in each structure and sufficient coverage across animals to allow for more detailed statistical comparisons (Figure 1D; for further details on the 619 verified electrode sites, see Figure S1).

### 3.2 Characterization of Antidyskinetic Effects of the Three Compounds

After drug injection, the severity of dyskinesia was quantified via manual scoring of the displayed AIMs (including orolingual, forelimb, and axial components) using a validated rating scale (Cenci and Lundblad 2007). The antidyskinetic effect of the three compounds was evaluated against treatment with L‐DOPA + vehicle (Figure 2A). When considering the whole recording period, all treatments showed an antidyskinetic effect on AIMs when compared to the vehicle condition (*p* < 0.001 for all conditions against vehicle; GLME and Tukey‐HSD test). Analyzing the period of peak dose dyskinesia (40–80 min after L‐DOPA injection; Skovgård et al. 2023), it was noted that a significant reduction of the global AIMs score, compared to vehicle treated recording sessions, was observed for amantadine and for mesdopetam 3 and 10 mg/kg (Figure 2B left panel; *p* < 0.001, *p* < 0.05, and *p* < 0.05, respectively; GLME and Tukey‐HSD test; for details on subcategories of AIMs, see Figure S3A). However, when analyzing the temporal profiles of the global AIMs score, it was evident that mesdopetam had a delayed antidyskinetic effect compared to amantadine and pimavanserin (Figure 2A). We therefore performed statistical comparisons of the treatments in a second time window 120–160 min after L‐DOPA injection. In this late phase, mesdopetam (3 and 10 mg/kg) and amantadine showed a highly significant antidyskinetic effect compared to vehicle treatment (Figure 2B right panel; *p* < 0.001; GLME and Tukey‐HSD test; it should be noted that in the current design, amantadine was injected 30 min earlier than mesdopetam, possibly explaining the temporal difference observed).

In addition to AIMs, L‐DOPA also induced hyperkinesia, which in the unilateral 6‐OHDA model is typically manifested as excessive rotational behavior directed towards the side contralateral to the lesion (as opposed to an ipsilateral side‐bias in spontaneous turning under baseline conditions, Figure S2B; Ungerstedt and Arbuthnott 1970; Björklund and Dunnett 2019). Analyzing the entire recording period, a significant reduction of contralateral rotational behavior compared to vehicle treatment was observed for high‐dose mesdopetam and amantadine (*p* < 0.001; GLME and Tukey‐HSD test). On the other hand, when breaking down the analysis of treatment effects into peak and late periods, as for AIMs, none of the treatments was found to result in statistically significant reductions in rotational behavior compared to vehicle. It is also noteworthy that the degree of contralateral rotational behavior induced by L‐DOPA throughout the recordings could not be related to the severity of concurrent AIMs in a straightforward manner (Figure S3B; cf. Figure 2C,D), confirming earlier reports pointing to rotations as a behavior loosely connected to AIMs (Cenci and Crossman 2018).

### 3.3 Neurophysiological Activity Signatures of LID

**FIGURE 3:**
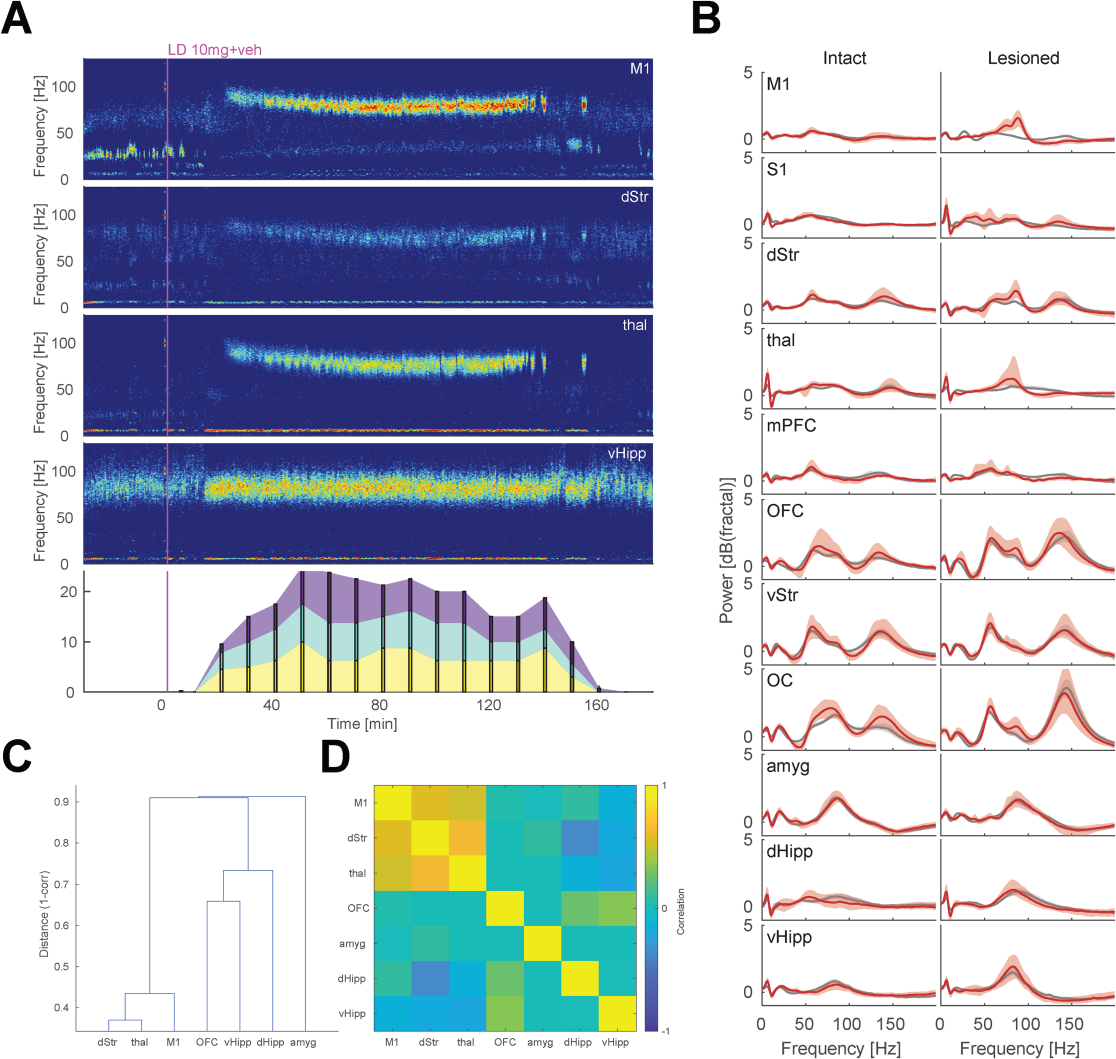
LFP oscillatory activity associated with dyskinesia. (A) Spectrograms from sensorimotor structures (M1, dStr, and thal) and ventral hippocampus from an example L‐DOPA + saline recording. Bottom panel depicts severity of the simultaneously displayed abnormal involuntary movements (AIMs; purple: orolingual, turquoise: axial, and yellow: forelimb). Note that relatively higher resemblance of narrowband gamma oscillations (NBGs) in M1, dStr, and thal, compared to vHipp. (B) Spectra from the intact and lesion hemisphere obtained by averaging all differential LFP signals from electrode pairs in the corresponding structures, across all animals and recordings. Grey: baseline. Red: L‐DOPA + vehicle. All spectra are normalized to the aperiodic component of the spectral power distributions to emphasize oscillatory activity. (C) Temporal correlations of NBG features (peak frequency/height/width) in all L‐DOPA recordings. (D) Relative difference between NBG features detected in different structures (distance corresponds to 1‐r, for the correlation values for the seven structures where NBG was detected in at least 5% of the sample periods). Panels (C) and (D) represent data from the lesioned hemisphere.

To characterize drug‐induced changes in network activity in different parts of the brain, local field potentials (LFPs) were constructed from differential voltage measurements obtained from all pairs of electrode tips located in the same brain structure (this practice of extracting LFP signals minimizes any potential contributions from current sources located outside the recorded structure). In agreement with previous studies in rat models of PD (Halje et al. 2012; Dupre et al. 2015; Tamte et al. 2016; Güttler et al. 2021; Skovgård et al. 2023) and in PD patients (Swann et al. 2016; Olaru et al. 2024), the expression of LID was found to be strongly associated with rhythmic synchronized transmembrane currents in parts of the cortico‐basal ganglia‐thalamic loop, reflected as distinct LFP oscillations appearing in a narrow part of the gamma band (Petersson et al. 2019, 2020; Halje et al. 2019). To further emphasize the oscillatory components in the analyzed power density spectra, we normalized all spectra to the corresponding nonperiodic background distributions (Wen and Liu 2016). The resulting normalized spectra revealed that narrowband gamma oscillations (NBGs), appearing in the range of 65–110 Hz, were particularly increased in M1, dStr, and thal during dyskinesia, but that oscillations in the same range could also be found in some limbic structures, e.g., vHipp, although not exclusively during periods of dyskinesia (Figure 3A). Furthermore, in agreement with previous reports (Tamte et al. 2016), NBGs in cortico‐basal ganglia‐thalamic structures were found to have very similar appearance in the time‐frequency domain when detected in multiple structures concurrently (Figure 3A).

Pooling the normalized spectra from all animals for the lesioned versus intact hemisphere during baseline and during peak dyskinesia, respectively, it was noticeable that, in addition to NBGs, several other oscillatory components were present in the different structures. In the context of LID, theta oscillations, in particular, have attracted a certain interest (Alonso‐Frech et al. 2006). Indeed, theta oscillations (4–10 Hz) tended to become more pronounced during dyskinesia in S1, thal, and dHipp (Figure 3B). However, as previously reported in the unilateral 6‐OHDA rat model of LID (Tamte et al. 2016), theta oscillations were observed bilaterally, in contrast to levodopa‐induced NBGs, which were restricted to the lesioned hemisphere. The relevance of this difference between NBG and theta is uncertain, but since LID in the rat PD model consists primarily of unilateral AIMs, while hyperkinesia involves muscle activity on both sides of the body, the NBG and theta could possibly be associated primarily with each of these motor signs. In any case, since both human and rodent studies indicate that NBGs, overall, are the most reliable biomarker for LID (Petersson et al. 2020; Olaru et al. 2024), we focused here the further analyses of neurophysiological activity related to LID on NBGs in the lesioned hemisphere.

### 3.4 Neurophysiological Activity Signatures in Cognitive‐Limbic Structures

A novel aspect of the current study is that several cognitive‐limbic structures were recorded in parallel with sensorimotor structures, thus providing a broader perspective on the brain state associated with levodopa treatment and dyskinesia. As noted above, NBGs in sensorimotor structures showed the strongest association to LID and severity of AIMs. Nevertheless, since oscillatory activity in a spectral range very similar to sensorimotor NBGs was simultaneously detected in cognitive‐limbic structures, it is possible that these oscillations are part of the same oscillatory phenomenon. Hence, to determine the relative resemblance of the activity in this frequency band in different parts of the brain, we compared distinct features of the oscillations (peak frequency/height/width) over time in all recording sessions (in the structures where oscillations in the 65–110 Hz band were present; [7/11 structures]). This analysis indicated the coexistence of three different oscillatory phenomena. Interestingly, oscillations in dHipp/vHipp and OFC were found to be more closely related to each other and largely inversely correlated to the sensorimotor NBGs, whereas amygdala oscillations were not strongly correlated with either of these two groups (Figure 3C,D). Thus, the sensorimotor NBG is most likely a separate oscillatory phenomenon from the cognitive‐limbic oscillations, even though they are present at the same time and in a similar range of the frequency spectrum. This tentative division between parallel oscillatory phenomena needs to be considered when evaluating outcomes of experimental treatments.

### 3.5 Neurophysiological Treatment Effects of the Three Antidyskinetic Compounds

**FIGURE 4:**
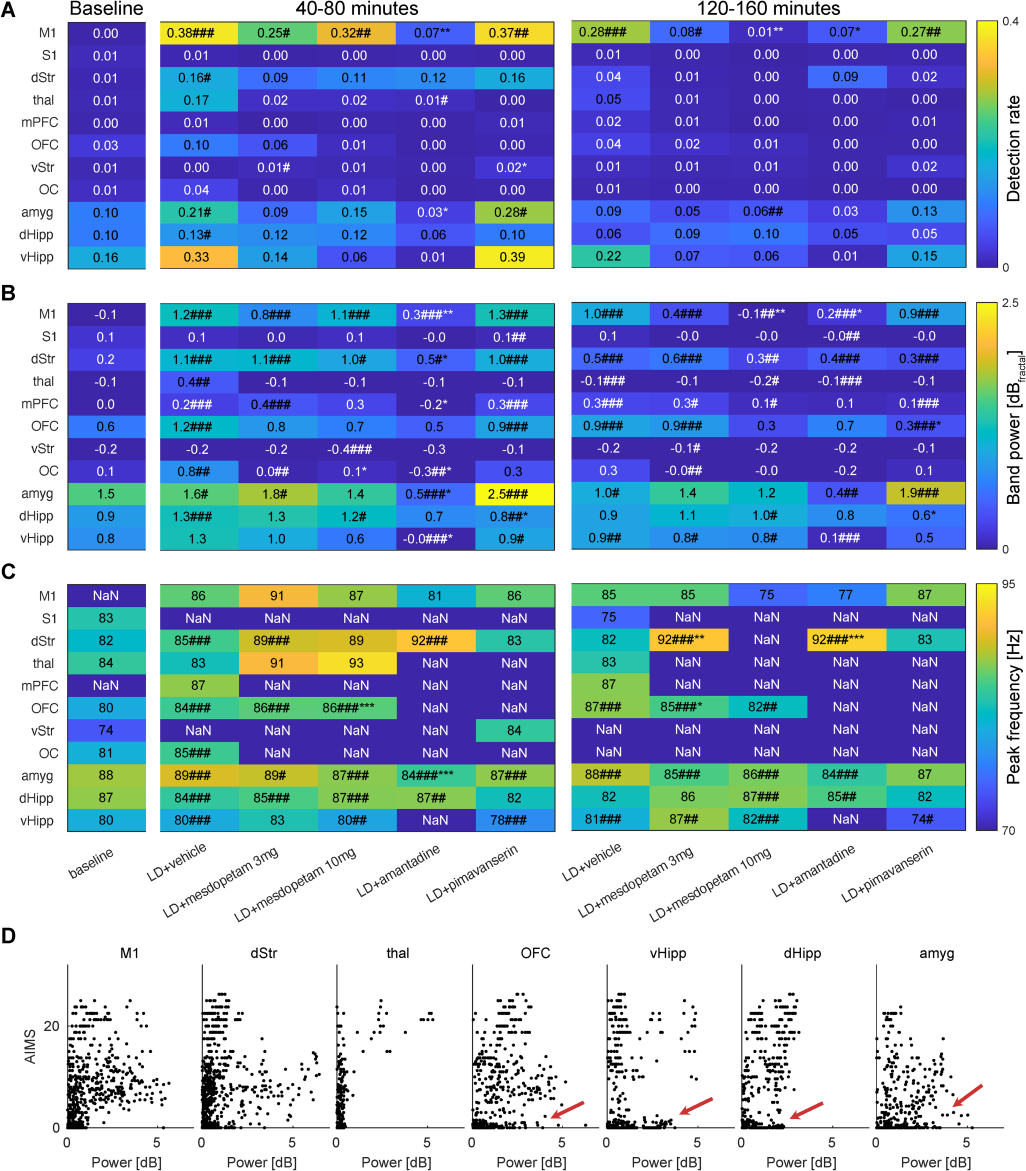
Summary of treatment effects on high‐gamma oscillation features. (A) Detection rate. (B) Band power (dB_fractal_). (C) Peak frequency (Hz). (D) Correlation between AIMs severity and the power of high‐gamma oscillations. Dark blue fields in Panel (C) denote structures where a distinct peak was absent. Significance is marked by [#], indicating a significant difference to baseline and [*] a significant difference to L‐DOPA + vehicle (ANOVA, Dunn's posttest). Red arrows in Panel (D) mark time periods where gamma oscillations were detected in the absence of AIMs. All data are from the lesioned hemisphere.

Next, to explore if the antidyskinetic effects observed in the behavioral assessments could be related to specific neurophysiological changes, we characterized how the different antidyskinetic compounds affected NBGs in the lesioned hemisphere. In specific, three features of the NBGs were analyzed in detail: (1) detection rate, (2) band power, and (3) peak oscillation frequency (for definitions, see Section 2.10). Interestingly, all three antidyskinetic compounds tended to alter these features in a graded manner in the different recorded structures. Both NBG detection rate and band power in sensorimotor structures were found to be partially reversed towards baseline conditions compared to L‐DOPA + vehicle by the different substances. In accordance with the temporal pattern of LID, these changes were faster for amantadine than for mesdopetam. Specifically, significant reductions in NBG band power, compared to L‐DOPA + vehicle treatment, were observed in M1 and dStr for amantadine 40–80 min after L‐DOPA injection, whereas a significant reduction of NBG by mesdopetam (observed in M1 at a dose of 10 mg/kg) was found in the 120–160‐min observation window (Figure 4A,B; [#] and [*] marks significant differences from baseline and L‐DOPA + vehicle, respectively, ANOVA, Dunn's posttest). Similar consistent changes could not be established for the NBG peak frequency, although a significant frequency increase was observed in the remaining dStr NBGs for both amantadine and mesdopetam in the late time window (it should be cautioned, however, that at lower detection rates, peak frequency estimates become less reliable; Figure 4C). Finally, changes in cognitive‐limbic structures were found to be less consistent. The most reliable treatment effect observed in the same frequency band was a reduction in all three features analyzed in amygdala for amantadine, together with a power decrease in dHipp for pimavanserin. Notably, when analyzing the correlation between AIMs severity and the power of gamma oscillations in all structures showing at least a 5% detection rate, it was clear that cognitive‐limbic structures, in contrast to sensorimotor structures, often showed gamma oscillations in the absence of AIMs (Figure 4D). However, in this context, it should also be acknowledged that since changes in oscillatory activity are not entirely limited to the frequency band that corresponds to NBGs in sensorimotor structures (65–110 Hz), a more appropriate comparison of the neurophysiological brain states induced by the different treatments should preferably include a broader frequency range (see Section 3.7 below).

### 3.6 Abnormally High Functional Connectivity Associated With LID Was Normalized by Antidyskinetic Treatment

**FIGURE 5:**
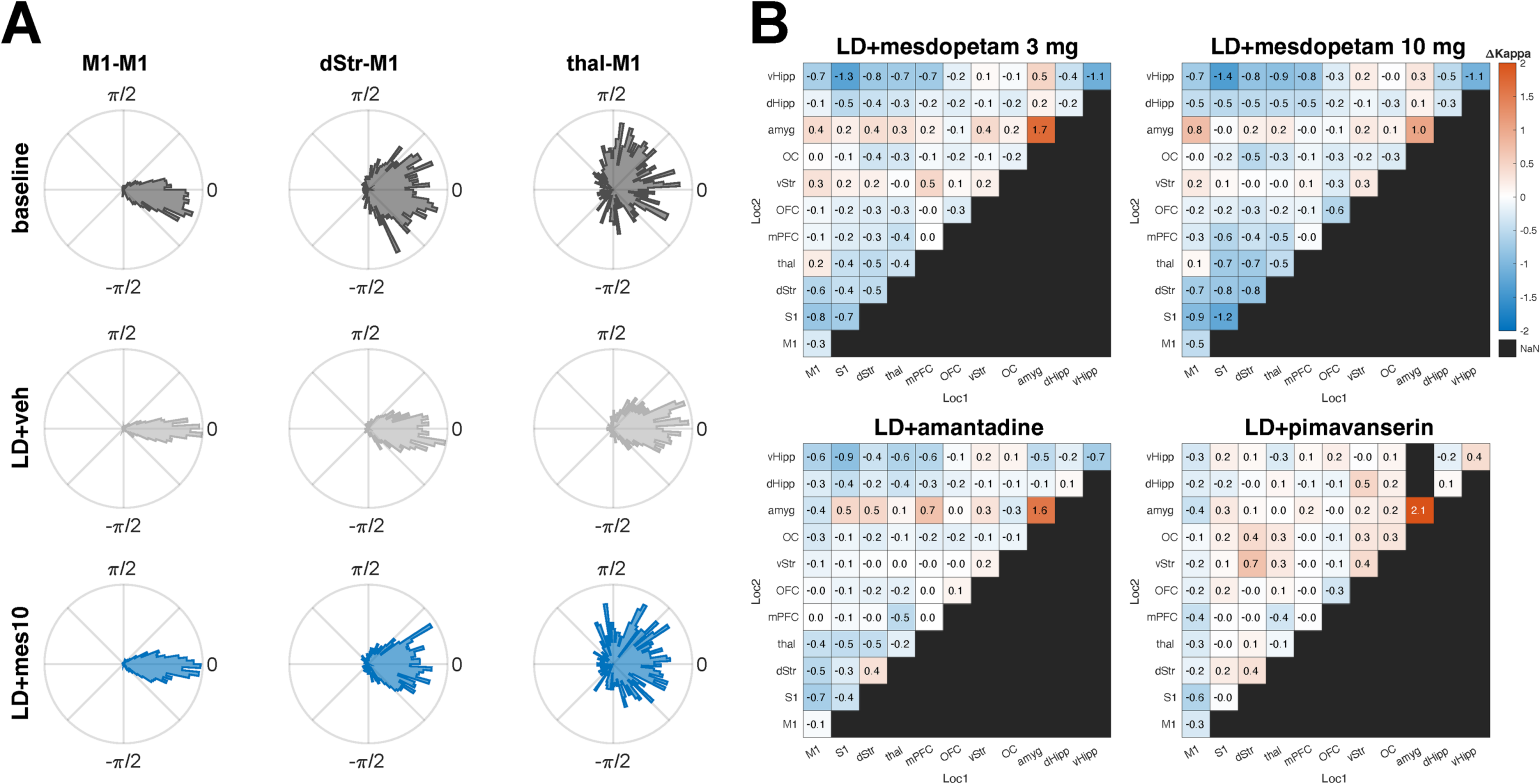
Antidyskinetic treatments alter functional connectivity in the high gamma band. (A) Distributions of phase relations between three example electrodes (located in M1, dStr, and thal) and a reference electrode located in M1, under three different treatment conditions. Note the narrower distributions induced by L‐DOPA and the partial reversal towards baseline by mesdopetam treatment. (B) Changes in phase‐synchronization (kappa‐values) for electrodes located in different pairs of structures (as indicated by the x‐ and y‐labels) for four treatment conditions compared to L‐DOPA + vehicle (cold colors denote reduced phase coupling). All data are from the lesioned hemisphere.

The finding that closely related NBGs are present simultaneously in M1, dStr, and thal during LID could be taken as an indication of an abnormally high degree of functional coupling within the sensorimotor cortico‐basal ganglia loop. Indeed, it has been hypothesized that increased functional coupling is a distinguishing pathophysiological feature of PD (Hirschmann et al. 2011; Santana et al. 2014; Petersson et al. 2020). To explore the degree of abnormal functional coupling, and how this coupling is affected by antidyskinetic treatments, we constructed a functional connectivity metric based on the consistency of phase relations of NBGs recorded concurrently by different electrodes in different structures (Stan et al. 2024). Example distributions of LFP phase relations between three different electrodes, located in M1, dStr, and thal, respectively, and a reference electrode located in M1 are shown for baseline, L‐DOPA + saline, and L‐DOPA + mesdopetam treatment (Figure 5A).

Two main effects are apparent. First, after L‐DOPA treatment, oscillations in M1, dStr, and thal became more coherent (i.e., the lobes denoting the phase distributions are narrower in the circular plots compared to baseline—here quantified through calculations of kappa‐values; defined as the inverse of the width of a circular normal distribution; σ^2^ = 1/κ). Second, after mesdopetam treatment, the distributions were partially reversed towards baseline distributions, indicating a step towards normalization from an exaggerated degree of functional coupling (LFPs were here measured in the late time window, 120–160 min after L‐DOPA injection, corresponding to the period of main antidyskinetic effect; cf. Figure 2). Using kappa‐values as a metric for functional coupling, we next analyzed the consistency of the phase relations between all pairs of recording electrodes. Interestingly, the pharmacological interventions did not only reduce sensorimotor coupling but also showed significant kappa reduction also between pairs of wires in cognitive‐limbic structures (Figure 5B; tables with the corresponding *p*‐values are appended as Data S1). In contrast, kappa‐values in amygdala showed a largely deviating pattern with increased kappa‐values across treatments. Finally, it is interesting to note the close correspondence between mesdopetam 3 and 10 mg/kg, indicating a high degree of reproducibility and analogous effects for doses in this range. Overall, the changes in kappa‐values observed in the 120–160‐min observation window largely appeared to correspond to the relative suppression of dyskinesia (cf. Figure 2), with a less pronounced effect by pimavanserin compared to mesdopetam and amantadine.

### 3.7 Characterization of LFP Changes Across the Broader Frequency Spectrum (1–200 Hz)

**FIGURE 6:**
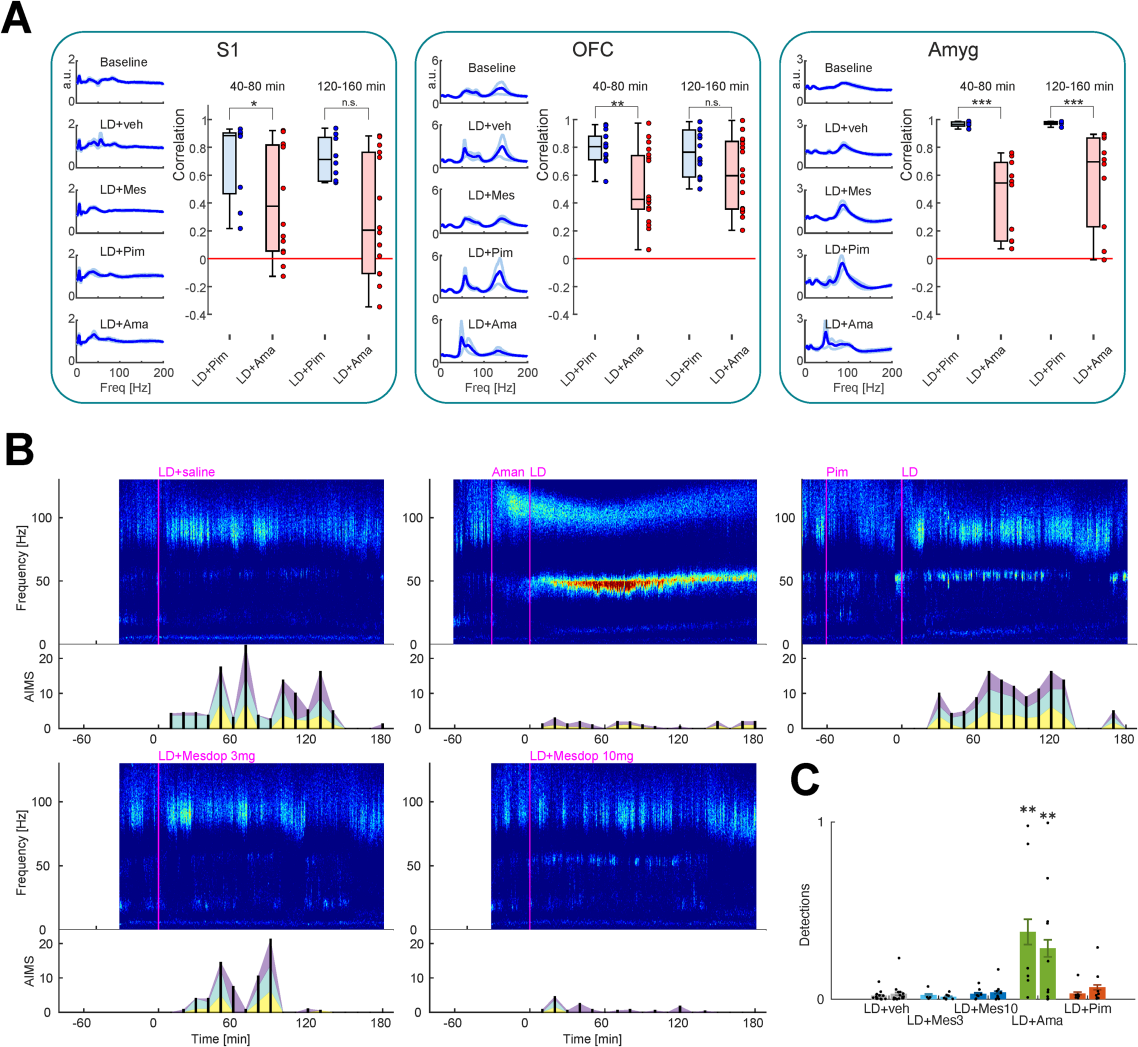
Oscillatory activity outside the high‐gamma band. (A) Comparative spectral analysis of mesdopetam vs. pimavanserin and amantadine effects for S1, OFC, and Amyg. Left: average normalized spectra of all animals (blue traces; light blue traces mark SE; arbitrary units) for different experimental conditions. Right: group data of pairwise spectral correlation coefficients between all individual mesdopetam recordings and the average amantadine/pimavanserin spectra in time windows 40–80 min and 120–160 min after L‐DOPA injection (Pearson's correlation coefficient). Data are presented as box plots [center line—median; edges—Q1/Q3; whiskers—nonoutlier extremes] with the individual values. * *p* < 0.05; ** *p* < 0.05; *** *p* < 0.001; n.s., not significant (two‐sided Wilcoxon ranksum test). (B) Spectrogram from amygdala recordings in an example rat under five different treatment conditions. Bottom panels depict severity of the simultaneously displayed AIMs (purple: orolingual, turquoise: axial, and yellow: forelimb). Note the low‐gamma oscillation visible in the amantadine + L‐DOPA condition. (C) The average detection rate of amygdalar low‐gamma oscillations, across all recordings, was significantly higher for amantadine than for any other treatment (*p* < 0.01; ANOVA, Dunn's posttest).

To assess potential mechanistic similarities between mesdopetam and existing treatments for dyskinesia and psychosis in PD, beyond their effects on gamma oscillations, we next compared the broader spectral profile of mesdopetam to amantadine and pimavanserin, respectively. Each compound was evaluated in combination with L‐DOPA, mimicking their intended clinical use. Specifically, for each of the 11 brain structures, the normalized spectral density distributions from each of the mesdopetam recordings were pairwise compared to the average, respective, spectrum of all amantadine and pimavanserin recording sessions. This resulted in a single spectral correlation value for each of these comparisons per structure. Interestingly, this analysis indicated that mesdopetam in most brain structures induced LFP changes more similar to pimavanserin than to amantadine (Figure 6A). In particular, a significantly higher spectral resemblance to pimavanserin compared to amantadine was found in S1, OFC, and amyg (*p* < 0.05, two‐sided Wilcoxon rank‐sum test; and a similar nonsignificant trend was observed in OC, dStr/vStr, and mPFC). This implies that while mesdopetam shares treatment effects with amantadine with respect to suppression of NBGs in sensorimotor structures, its broader spectral treatment profile closer resembles pimavanserin across several sensorimotor and cognitive‐limbic structures. Intriguingly, this difference was most noticeable for amygdala. A closer inspection of the average amantadine LFP power spectrum in amygdala revealed an evident difference in the peak frequency of the main oscillatory component, compared to both mesdopetam and pimavanserin (with a peak near 50 Hz rather than 80 Hz), pointing to a distinct oscillatory phenomenon in the lower range of the gamma band. Examining the temporal development of these amantadine‐induced oscillations suggested they arise as a consequence of the combined L‐DOPA + amantadine treatment (Figure 6B). Low‐gamma oscillations were not exclusively found in the amantadine treated condition, but pooled data from all animals showed that they were significantly more frequently detected in this condition than in any of the other treatments, including L‐DOPA + saline (Figure 6C; *p* < 0.01, ANOVA, Dunn's posttest), and were observed in both the lesioned and intact hemisphere.

## 4 Discussion

By performing multistructure neurophysiological recordings in parkinsonian and dyskinetic rats, we here characterized the changes in neuronal population activity in sensorimotor and in cognitive‐limbic brain structures associated with LID. We have also outlined how brain states are altered when L‐DOPA treatment is combined with the two clinically used compounds amantadine and pimavanserin or the novel prospective antidyskinetic and antipsychotic treatment mesdopetam. These analyses were performed during peak‐dose dyskinesia (40–80 min after L‐DOPA; Fabbrini et al. 2007; Skovgård et al. 2023) and, because a delayed effect of mesdopetam was observed, also 120–160 min after L‐DOPA. On a behavioral level, both amantadine and mesdopetam were found to reduce the expression of dyskinesia. While a tendency for an antidyskinetic effect of pimavanserin was observed in the time windows selected for neurophysiological analyses, this effect was not statistically significant compared to rats treated with L‐DOPA + vehicle. Hence, in this respect, our results do not lend support to the notion of antidyskinetic properties of pimavanserin. But given that other 5‐HT2A antagonists have been suggested to have certain antidyskinetic effects, and the current observation that pimavanserin, when evaluated over the entire recording period, showed significant reductions in AIMs, this hypothesis nevertheless deserves further exploration in future studies (Hamadjida et al. 2017). Regarding rotations, none of the treatments reached statistical significance compared to L‐DOPA + vehicle, in the time windows selected for neurophysiological analyses, indicating that the antidyskinetic treatment effects observed during peak‐dose dyskinesia are more selectively reducing AIMs and cannot be ascribed to a general suppression of motor behavior. This effect is in line with several earlier reports demonstrating that contralateral rotations, in contrast to AIMs, are less reliable as a specific measure of L‐DOPA‐induced motor complications in this model of LID (but may provide certain information on pharmacodynamics, for a review, see Cenci and Crossman 2018; see also Figure S3B). It should be noted, however, that for more detailed behavioral characterizations of antidyskinetic effects, larger sample sizes would clearly be an advantage.

In agreement with earlier studies, we found that suppression of NBGs in sensorimotor structures was strongly associated with reduction of dyskinesia (Halje et al. 2012; Swann et al. 2016; Petersson et al. 2019; Olaru et al. 2024). In particular, for both mesdopetam and amantadine, significant reductions in M1 NBGs were observed, closely matching the manually scored severity of the simultaneously displayed dyskinesia. It should be cautioned that a causal relationship between sensorimotor NBGs and dyskinesia has not been proven. Nevertheless, our results clearly further strengthen the case for NBGs as a reliable neurophysiological biomarker in drug development, at least in this animal model of LID. In contrast, we did not find strong evidence for an association between LID and gamma‐oscillations in cognitive‐limbic structures. Instead, oscillatory activity in the same frequency interval in dHipp/vHipp and OFC appeared to be inversely correlated with sensorimotor NBGs, and similar gamma activity in amygdala did not seem to be closely related to either of these two groups of structures. If parallel circuits maintain different gamma‐oscillations in sensorimotor and cognitive‐limbic structures in the L‐DOPA treated state, the reduced functional coupling induced by antidyskinetic treatments, as was demonstrated in the phase analyses, may represent differential effects on sensorimotor and cognitive‐limbic systems.

A limitation of the current study is that no behavioral tests designed to specifically assess cognitive‐limbic functions were performed (as LID largely precludes tests relying on volitional motor control). Consequently, the interpretation of cognitive‐limbic gamma in the current context becomes a bit speculative. One possibility is, for example, that increased gamma outside sensorimotor circuits is related to compensatory mechanisms being engaged to suppress involuntary behaviors and thoughts (see, e.g., Guan et al. 2022). However, brain circuit oscillations include a wide variety of phenomena, where excessive synchronous activity often is thought to have detrimental effects (Halje et al. 2019). Indeed, recent evidence suggests that the coupling between excessive oscillation components at different frequencies in cognitive‐limbic circuits could potentially be used as a biomarker to guide neuromodulatory therapy, for example, in depression (Young et al. 2024). Consequently, we propose that the further clarification of the role of these widely distributed, yet apparently spatially separated, oscillatory phenomena will be of key importance to identify both the pathophysiology underlying symptoms in various disorders, as well as to illuminate treatment mechanisms for drugs designed to target certain categories of symptoms.

Beyond the investigation of pharmacological effects on specific neurophysiological biomarkers of disease, neuronal population activity can also inform on other physiological changes induced by a drug. For example, while the antidyskinetic effects of pimavanserin were found to be less pronounced, pimavanserin is primarily used for the treatment of PD psychosis, making it highly relevant as a reference drug for characterization, neurophysiological effects induced in cognitive‐limbic structures in parkinsonian levodopa‐treated animals (Stan et al. 2024). Conversely, amantadine suppresses dyskinesia, but side‐effects often occur in the psychiatric domain, which may be related to recognizable deviating activity patterns in discrete brain structures. We here identified oscillations in the lower gamma band in amygdala as a particularly conspicuous phenomenon in amantadine treated rats. The functional meaning of these oscillations remains to be clarified, and to our knowledge, amygdalar low‐gamma oscillations have not been reported in a similar context before. Nonetheless, it is tempting to speculate that some neuropsychiatric side effects of amantadine could be related to this aberrant activity pattern (Schwab et al. 1969; Pahwa et al. 2015).

Finally, our current results suggest that although mesdopetam resembles amantadine in its ability to suppress sensorimotor NBGs, the overall similarity taking the broader spectral changes into account suggests a close functional relationship also to pimavanserin, perhaps giving clues to the antipsychotic‐like properties displayed by mesdopetam (Waters et al. 2020; Stan et al. 2024).

## Author Contributions

**Abdolaziz Ronaghi:** data curation, investigation, visualization, writing – review and editing. **Tiberiu Loredan Stan:** data curation, investigation, visualization, writing – review and editing. **Sebastian A. Barrientos:** data curation, formal analysis, software, visualization, writing – review and editing. **Pär Halje:** data curation, formal analysis, methodology, resources, visualization, writing – review and editing. **Azat Nasretdinov:** data curation, formal analysis, software, visualization, writing – review and editing. **Luciano Censoni:** data curation, formal analysis, software, visualization, writing – review and editing. **Sebastian Sulis Sato:** data curation, investigation, writing – review and editing. **Evgenya Malinina:** data curation, investigation, writing – review and editing. **Joakim Tedroff:** conceptualization, funding acquisition, writing – review and editing. **Nicholas Waters:** conceptualization, funding acquisition, resources, writing – review and editing. **Per Petersson:** conceptualization, funding acquisition, methodology, project administration, supervision, writing – original draft, writing – review and editing.

## Conflicts of Interest

The authors declare the following financial interests/personal relationships that may be considered as potential competing interests: Joakim Tedroff and Nicholas Waters are employed by IRL AB and hold stock in IRL AB. The remaining authors declare no conflicts of interest.

### Peer Review

The peer review history for this article is available at https://www.webofscience.com/api/gateway/wos/peer‐review/10.1111/ejn.70032.

## Supporting information


**Data S1.** Statistical significance levels for phase analysis in main Figure 5.
**Figure S1.** Overview of electrode locations.
**Figure S2.** Assessment of 6‐OHDA lesion severity.
**Figure S3.** Quantification of abnormal involuntary movements involving different muscle groups.

## Data Availability

Data are available on reasonable request.
