# Supplementary material for: Neurophysiological Treatment Effects of Mesdopetam, Pimavanserin and Amantadine in a Rodent Model of Levodopa‐Induced Dyskinesia
Source: Eur J Neurosci. 2025 Mar 5;61(5):e70032. doi: 10.1111/ejn.70032 (PMC11881547; doi:10.1111/ejn.70032)
Supplement: Supplementary file 1 — Data S1. Statistical significance levels for phase analysis in main Figure 5. Figure S1. Overview of electrode locations. Figure S2. Assessment of 6‐OHDA lesion severity. Figure S3. Quantification of abnormal involuntary movements involving different muscle groups. [file EJN-61-0-s001.pdf]

Supplementary Data - Statistical significance levels for phase analysis in main figure 5

Loc1	Loc2	k	p	H	n
M1	M1	-0,296009944	0,472362281		119
M1	OC	0,017697359	0,902313544		87
M1	OFC	-0,088160636	0,138640711		132
M1	S1	-0,787170559	2,76585E-05	***	79
M1	amyg	0,411805223	6,3127E-08	***	84
M1	dHipp	-0,149657134	0,017899518	*	77
M1	dStr	-0,565135231	4,23441E-17	***	173
M1	mPFC	-0,121892147	0,024508851	*	82
M1	thal	0,224019418	0,05913208		177
M1	vHipp	-0,661111981	1,1057E-05	***	53
M1	vStr	0,341119208	5,82084E-06	***	55
OC	OC	-0,162244841	0,506267371		71
OC	amyg	0,152515963	5,45548E-06	***	38
OC	dHipp	-0,235814125	3,10187E-07	***	104
OC	vHipp	-0,061147295	0,139078913		39
OFC	OC	-0,17709091	0,078979473		47
OFC	OFC	-0,257800189	0,447623636		37
OFC	amyg	-0,105091901	0,004245441	**	36
OFC	dHipp	-0,224848034	0,002562049	**	48
OFC	vHipp	-0,197019597	0,001425648	**	33
OFC	vStr	0,077386045	0,819868406		28
S1	OC	-0,072585771	0,004137208	**	21
S1	OFC	-0,194239056	0,612050976		49
S1	S1	-0,703886763	0,002624512	**	16
S1	amyg	0,192554094	0,002767775	**	58
S1	dHipp	-0,530810768	0,001731827	**	25
S1	dStr	-0,440812325	2,64299E-07	***	100
S1	mPFC	-0,243653778	0,016812693	*	35
S1	thal	-0,423289407	7,16408E-06	***	77
S1	vHipp	-1,292324093	3,16517E-06	***	29
S1	vStr	0,160082169	0,018066406	*	15
amyg	amyg	1,722205702	1,47113E-10	***	93
amyg	dHipp	0,155579356	0,097989303		16
amyg	vHipp	0,488921747	0,0078125	**	8
dHipp	dHipp	-0,166329584	0,829013032		30
dHipp	vHipp	-0,409618928	0,125705641		37
dStr	OC	-0,360428782	9,86526E-06	***	86
dStr	OFC	-0,253173334	3,65645E-06	***	97
dStr	amyg	0,387727444	1,82628E-11	***	151
dStr	dHipp	-0,412967775	1,17164E-09	***	85
dStr	dStr	-0,48972046	0,025667496	*	133
dStr	mPFC	-0,275230372	9,94711E-05	***	77
dStr	thal	-0,488929208	5,54793E-18	***	188
dStr	vHipp	-0,755914647	2,23384E-12	***	71
dStr	vStr	0,186377731	0,377742804		48
mPFC	OC	-0,063273137	0,587890625		13
mPFC	OFC	-0,02488694	0,345773558		54
mPFC	amyg	0,150217887	6,68817E-07	***	42
mPFC	dHipp	-0,171687783	0,073450861		21

mPFC	mPFC	0,012532734	0,807739258		14
mPFC	vHipp	-0,721135988	0,00193099	**	17
mPFC	vStr	0,462281491	0,009765625	**	11
thal	OC	-0,318706525	2,64859E-05	***	146
thal	OFC	-0,25129593	1,61379E-10	***	102
thal	amyg	0,333741808	2,9085E-07	***	109
thal	dHipp	-0,336472878	7,00612E-07	***	105
thal	mPFC	-0,436951524	0,009369031	**	54
thal	thal	-0,383913364	0,018883523	*	95
thal	vHipp	-0,728835633	2,24106E-06	***	68
thal	vStr	-0,024525559	0,487704864		82
vHipp	vHipp	-1,11097305	0,052246094		12
vStr	OC	-0,069905834	0,363352799		79
vStr	amyg	0,436209225	1,15759E-08	***	45
vStr	dHipp	-0,0939984	0,036147227	*	47
vStr	vHipp	0,07327887	0,234320972		16
vStr	vStr	0,173380399	0,876721743		16

Loc1	Loc2	k	p	H	n
M1	M1	-0,476776513	0,962401931		120
M1	OC	-0,026160288	0,365078057		87
M1	OFC	-0,170261736	0,059338925		130
M1	S1	-0,939005592	1,87022E-05	***	73
M1	amyg	0,760863578	3,73102E-13	***	84
M1	dHipp	-0,461648735	0,003061985	**	76
M1	dStr	-0,688446269	1,2152E-20	***	173
M1	mPFC	-0,279405219	0,027067848	*	81
M1	thal	0,085142915	0,7370521		175
M1	vHipp	-0,703420903	2,03912E-06	***	53
M1	vStr	0,234396154	0,00643503	**	63
OC	OC	-0,278186025	0,197324025		71
OC	amyg	0,149813469	9,15325E-07	***	38
OC	dHipp	-0,259106137	3,31636E-06	***	104
OC	vHipp	-0,008363825	0,459533704		39
OFC	OC	-0,267224456	0,02626619	*	47
OFC	OFC	-0,562686426	0,019713757	*	33
OFC	amyg	-0,056091762	0,031369896	*	36
OFC	dHipp	-0,235258985	6,20641E-07	***	48
OFC	vHipp	-0,255571969	3,01755E-05	***	33
OFC	vStr	-0,256680062	0,020674093	*	33
S1	OC	-0,182032511	0,000418868	***	21
S1	OFC	-0,204379806	6,18179E-06	***	42
S1	S1	-1,222378967	0,006713867	**	15
S1	amyg	-0,016772302	0,256688027		58
S1	dHipp	-0,516415292	2,3518E-05	***	24
S1	dStr	-0,775086955	1,47621E-11	***	99
S1	mPFC	-0,56267844	1,62342E-05	***	31
S1	thal	-0,730389985	1,46826E-08	***	75
S1	vHipp	-1,370032359	2,8489E-06	***	29
S1	vStr	0,069113504	0,325870024		16
amyg	amyg	1,046502686	5,81106E-09	***	93
amyg	dHipp	0,140952608	0,133729893		16
amyg	vHipp	0,296818247	0,078125		8
dHipp	dHipp	-0,312246978	0,011748106	*	30
dHipp	vHipp	-0,513882397	0,078823718		37
dStr	OC	-0,53161327	7,89154E-13	***	86
dStr	OFC	-0,318461562	3,52973E-10	***	97
dStr	amyg	0,17951389	4,39346E-07	***	151
dStr	dHipp	-0,502791811	1,01104E-10	***	85
dStr	dStr	-0,82808276	3,57434E-05	***	133
dStr	mPFC	-0,400249574	5,57055E-10	***	77
dStr	thal	-0,725269594	4,15996E-22	***	188
dStr	vHipp	-0,839984842	1,80361E-11	***	71
dStr	vStr	-0,003563562	0,807455321		51
mPFC	OC	-0,110748516	0,146484375		13
mPFC	OFC	-0,126064722	0,023809944	*	54
mPFC	amyg	-0,04818705	0,935224198		42
mPFC	dHipp	-0,474755076	0,001865752	**	21

mPFC	mPFC	-0,033104125	0,583007813		14
mPFC	vHipp	-0,797321765	0,000420791	***	17
mPFC	vStr	0,115964223	0,846923828		15
thal	OC	-0,331143854	4,14525E-09	***	146
thal	OFC	-0,165968859	3,27156E-09	***	102
thal	amyg	0,217651998	2,87655E-05	***	109
thal	dHipp	-0,477691865	5,15842E-09	***	105
thal	mPFC	-0,483408662	0,001648659	**	54
thal	thal	-0,473350746	0,00786884	**	95
thal	vHipp	-0,876828748	5,40215E-07	***	68
thal	vStr	-0,043429979	0,04304771	*	82
vHipp	vHipp	-1,065722932	0,042480469	*	12
vStr	OC	-0,177388069	0,002651029	**	79
vStr	amyg	0,248795363	2,54145E-08	***	45
vStr	dHipp	-0,115342486	0,006568092	**	48
vStr	vHipp	0,210866545	0,000531243	***	16
vStr	vStr	0,259055173	0,836138662		16

Loc1	Loc2	k	p	H	n
M1	M1	-0,052358681	0,945713516		120
M1	OC	-0,301180006	3,66373E-08	***	87
M1	OFC	-0,042739583	0,190210339		141
M1	S1	-0,676511577	4,54185E-09	***	73
M1	amyg	-0,372092426	1,1694E-07	***	84
M1	dHipp	-0,253172325	0,009829859	**	87
M1	dStr	-0,536435338	8,49797E-19	***	168
M1	mPFC	0,010013097	0,422113526		92
M1	thal	-0,434659055	8,46733E-18	***	170
M1	vHipp	-0,612489471	7,90859E-08	***	53
M1	vStr	-0,146161767	0,000277677	***	63
OC	OC	-0,13841045	0,040239567	*	71
OC	amyg	-0,278598277	2,52639E-06	***	38
OC	dHipp	-0,10418814	5,11957E-06	***	103
OC	vHipp	0,131724907	0,012361866	*	38
OFC	OC	-0,189407068	0,189456818		47
OFC	OFC	0,12642852	0,386923609		39
OFC	amyg	0,040087154	0,441407759		36
OFC	dHipp	-0,167159027	0,000205258	***	58
OFC	vHipp	-0,058804355	0,140455771		33
OFC	vStr	-0,008181389	0,743227084		35
S1	OC	-0,123309848	0,00061791	***	21
S1	OFC	-0,140179447	0,047263339	*	44
S1	S1	-0,399838637	0,063720703		15
S1	amyg	0,456047876	0,010502025	*	58
S1	dHipp	-0,440105762	0,000526647	***	26
S1	dStr	-0,256260168	0,215325919		99
S1	mPFC	-0,0711275	0,190559491		33
S1	thal	-0,473423195	2,40498E-07	***	75
S1	vHipp	-0,881418707	6,04355E-05	***	29
S1	vStr	-0,064218477	0,876721743		16
amyg	amyg	1,58418246	7,61768E-05	***	93
amyg	dHipp	-0,114782822	0,605094946		16
amyg	vHipp	-0,493114302	0,25		8
dHipp	dHipp	0,075215952	0,935914967		33
dHipp	vHipp	-0,217337409	0,9939815		37
dStr	OC	-0,228148246	4,03503E-06	***	86
dStr	OFC	-0,176614342	0,001737711	**	96
dStr	amyg	0,453487022	1,00757E-16	***	151
dStr	dHipp	-0,249862478	2,34877E-07	***	89
dStr	dStr	0,351882771	0,000304632	***	132
dStr	mPFC	-0,032958154	0,787692371		78
dStr	thal	-0,453147922	1,04165E-17	***	186
dStr	vHipp	-0,403753226	7,84863E-06	***	71
dStr	vStr	-0,02771497	0,822007899		51
mPFC	OC	-0,19112779	0,032714844	*	13
mPFC	OFC	0,014278789	0,250799319		67
mPFC	amyg	0,70418368	6,8218E-05	***	42
mPFC	dHipp	-0,265628239	0,047578509	*	31
mPFC	mPFC	0,005167682	0,846923828		20
mPFC	vHipp	-0,560176378	0,000293053	***	17
mPFC	vStr	-0,021362834	0,917631593		17
thal	OC	-0,120510063	2,82429E-05	***	146
thal	OFC	-0,188689793	6,4611E-05	***	96
thal	amyg	0,110382078	0,019865635	*	109
thal	dHipp	-0,380589514	1,60249E-10	***	104

thal	mPFC	-0,513219526	0,005773518	**	50
thal	thal	-0,215837863	0,685932774		95
thal	vHipp	-0,596243758	0,00065431	***	68
thal	vStr	0,009720342	0,304757452		82
vHipp	vHipp	-0,698109514	0,063964844		12
vStr	OC	-0,135405059	0,127317548		79
vStr	amyg	0,316032455	0,002727894	**	45
vStr	dHipp	-0,067825397	0,002993709	**	50
vStr	vHipp	0,175155647	0,034001025	*	16
vStr	vStr	0,15234022	0,147660256		16

Loc1	Loc2	k	p	H	n
M1	M1	-0,305226619	0,944348234		99
M1	OC	-0,06963571	0,034309447	*	45
M1	OFC	-0,179009055	0,000386521	***	127
M1	S1	-0,602795085	3,45044E-06	***	59
M1	amyg	-0,419819484	5,382E-10	***	56
M1	dHipp	-0,22402055	0,074859809		59
M1	dStr	-0,168188006	9,91423E-05	***	147
M1	mPFC	-0,422759121	2,40245E-13	***	77
M1	thal	-0,269342066	7,10098E-11	***	128
M1	vHipp	-0,314059119	0,005254554	**	39
M1	vStr	-0,15136658	0,006788542	**	42
OC	OC	0,306465182	0,001347184	**	56
OC	amyg	0,237278123	0,153076172		14
OC	dHipp	0,183275303	3,94939E-06	***	80
OC	vHipp	0,11132895	0,142777753		27
OFC	OC	-0,143098509	0,053268374		35
OFC	OFC	-0,329080825	0,658258089		38
OFC	amyg	-0,002634804	0,7326735		28
OFC	dHipp	-0,140578825	0,004215403	**	48
OFC	vHipp	0,187582969	0,00316166	**	29
OFC	vStr	-0,087533747	0,198240555		29
S1	OC	0,157644184	0,8203125		9
S1	OFC	0,172784795	0,039732566	*	40
S1	S1	-0,029966101	0,241210938		14
S1	amyg	0,255560633	0,005680697	**	50
S1	dHipp	-0,189733654	0,327143875		18
S1	dStr	0,22882389	4,20095E-07	***	93
S1	mPFC	-0,002507085	0,585711569		30
S1	thal	-0,012508369	0,98907554		63
S1	vHipp	0,174063515	0,798247624		25
S1	vStr	0,077575522	0,6953125		10
amyg	amyg	2,126479292	1,77431E-12	***	87
dHipp	dHipp	0,066170274	0,548089799		27
dHipp	vHipp	-0,194959044	0,837243675		29
dStr	OC	0,412872367	2,46404E-06	***	68
dStr	OFC	-0,021997384	0,157266028		90
dStr	amyg	0,073473455	7,98186E-06	***	139
dStr	dHipp	-0,040319791	0,053362445		77
dStr	dStr	0,351240513	6,30119E-08	***	129
dStr	mPFC	-0,001197654	0,928456403		72
dStr	thal	0,134407746	4,82285E-06	***	168
dStr	vHipp	0,081520427	0,934895857		65
dStr	vStr	0,70731962	0,00139983	**	42
mPFC	OC	-0,044571363	0,8125		7
mPFC	OFC	-0,008094645	0,243928587		58
mPFC	amyg	0,163072552	0,00031358	***	38
mPFC	dHipp	-0,095308885	0,002030638	**	24
mPFC	mPFC	-0,011168073	0,977966309		15
mPFC	vHipp	0,091865924	0,890380859		15

mPFC	vStr	-0,00987862	0,587890625		13
thal	OC	0,261150853	4,58111E-09	***	110
thal	OFC	0,083183118	0,017663311	*	84
thal	amyg	0,010098881	0,119300415		85
thal	dHipp	0,101777746	0,091354981		80
thal	mPFC	-0,358502047	0,00509688	**	44
thal	thal	-0,107572794	0,874238155		80
thal	vHipp	-0,273695958	0,020973849	*	56
thal	vStr	0,255098784	0,000185258	***	64
vHipp	vHipp	0,394137121	0,764648438		11
vStr	OC	0,290097083	0,000142793	***	61
vStr	amyg	0,173652621	3,26327E-05	***	33
vStr	dHipp	0,470120997	0,000637151	***	38
vStr	vHipp	-0,004665717	0,845703125		10
vStr	vStr	0,418377994	0,057373047		13

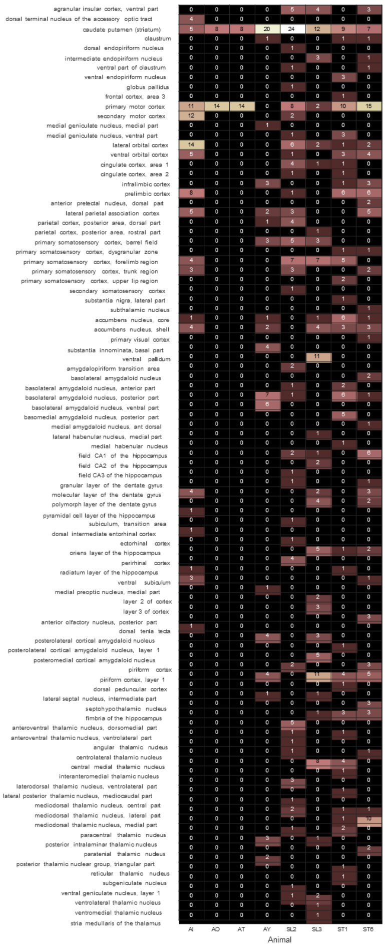

Functional grouping LID

PaxinosFull	Paxinos	GroupFull	Group
agranular insular cortex, dorsal part	AID	agranular insular cortex	AI
agranular insular cortex, posterior part	AIP	agranular insular cortex	AI
agranular insular cortex, ventral part	AIV	agranular insular cortex	AI
caudate putamen (striatum)	CPu	dorsal striatum	CPu
claustrum	Cl	claustrum	Cl
ventral part of claustrum	VCl	claustrum	Cl
globus pallidus	GP	globus pallidus	GP
nucleus of the brachium of the inferior colliculus	BIC	inferior colliculus	IC
external cortex of the inferior colliculus	ECIC	inferior colliculus	IC
brachium of the inferior colliculus	bic	inferior colliculus	IC
primary motor cortex	M1	primary motor cortex	M1
secondary motor cortex	M2	secondary motor cortex	M2
medial geniculate nucleus, dorsal part	MGD	medial geniculate nucleus	MG
medial geniculate nucleus, medial part	MGM	medial geniculate nucleus	MG
medial geniculate nucleus, ventral part	MGV	medial geniculate nucleus	MG
medial preopFc area	MPA	preopFc area	MPA
lateral orbital cortex	LO	orbitofrontal cortex	OFC
ventral orbital cortex	VO	orbitofrontal cortex	OFC
infralimbic cortex	IL	prefrontal cortex	PFC
prelimbic cortex	PrL	prefrontal cortex	PFC
cingulate cortex, area 1	Cg1	prefrontal cortex	PFC
cingulate cortex, area 2	Cg2	prefrontal cortex	PFC
anterior pretectal nucleus	APT	pretectum	PT
anterior pretectal nucleus, dorsal part	APTD	pretectum	PT
lateral parietal associaFon cortex	LPtA	parietal associaFon cortex	PtA
parietal cortex, posterior area, dorsal part	PtPD	parietal associaFon cortex	PtA
parietal cortex, posterior area, rostral part	PtPR	parietal associaFon cortex	PtA
retrosplenial granular cortex, c region	RSGc	parietal associaFon cortex	PtA
primary somatosensory cortex, barrel field	S1BF	primary somatosensory cortex	S1
primary somatosensory cortex, dysgranular zone	S1DZ	primary somatosensory cortex	S1
primary somatosensory cortex, forelimb region	S1FL	primary somatosensory cortex	S1
primary somatosensory cortex, trunk region	S1Tr	primary somatosensory cortex	S1
primary somatosensory cortex, upper lip region	S1ULp	primary somatosensory cortex	S1
secondary somatosensory cortex	S2	secondary somatosensory cortex	S2
intermediate gray layer of the superior colliculus	InG	superior colliculus	SC
intermediate white layer of the superior colliculus	InWh	superior colliculus	SC
brachium of the superior colliculus	bsc	superior colliculus	SC
substanFa nigra, lateral part	SNL	substanFa nigra, reFcular part	SNR
substanFa nigra, reFcular part	SNR	substanFa nigra, reFcular part	SNR
accumbens nucleus, core	AcbC	ventral striatum	StrV
accumbens nucleus, shell	AcbSh	ventral striatum	StrV
islands of Calleja	ICj	ventral striatum	StrV
lateral accumbens shell	LAcbSh	ventral striatum	StrV
secondary visual cortex, lateral area	V2L	secondary visual cortex	V2
ventral pallidum	VP	ventral pallidum	VP
amygdalohippocampal area, anterolateral part	AHiAL	amygdala	amyg
amygdalohippocampal area, posterolateral	AHiPL	amygdala	amyg
amygdalohippocampal area, posteromedial part	AHiPM	amygdala	amyg
amygdalopiriform transiFon area	APir	amygdala	amyg
basolateral amygdaloid nucleus, anterior part	BLA	amygdala	amyg
basolateral amygdaloid nucleus, posterior part	BLP	amygdala	amyg
basolateral amygdaloid nucleus, ventral part	BLV	amygdala	amyg
basomedial amygdaloid nucleus, posterior part	BMP	amygdala	amyg
intersFFal nucleus of the posterior limb of the anterior commissure	IPAC	amygdala	amyg
lateral amygdaloid nucleus, ventromedial part	LaVM	amygdala	amyg
bed nucleus of the stria terminalis, intraamygdaloid division	STIA	amygdala	amyg
lateral habenular nucleus, medial part	LHbM	habenula	habenula
medial habenular nucleus	MHb	habenula	habenula

PaxinosFull	Paxinos	GroupFull	Group
field CA2 of the hippocampus	CA2	dorsal hippocampus	hippD
granular layer of the dentate gyrus	GrDG	dorsal hippocampus	hippD
lacunosum moleculare layer of the hippocampus	LMol	dorsal hippocampus	hippD
molecular layer of the dentate gyrus	MoDG	dorsal hippocampus	hippD
stratum lucidum of the hippocampus	SLu	dorsal hippocampus	hippD
subiculum, transiFon area	STr	dorsal hippocampus	hippD
field CA1 of the hippocampus	CA1	ventral hippocampus	hippV
field CA3 of the hippocampus	CA3	ventral hippocampus	hippV
ectorhinal cortex	Ect	ventral hippocampus	hippV
oriens layer of the hippocampus	Or	ventral hippocampus	hippV
perirhinal cortex	PRh	ventral hippocampus	hippV
polymorph layer of the dentate gyrus	PoDG	ventral hippocampus	hippV
radiatum layer of the hippocampus	Rad	ventral hippocampus	hippV
ventral intermediate entorhinal cortex	VIEnt	ventral hippocampus	hippV
ventral subiculum	VS	ventral hippocampus	hippV
lateral olfactory tract	lo	olfactory tract	lo
medial forebrain bundle	mX	medial forebrain bundle	mX
layer 2 of cortex	2	olfactory cortex	olf
layer 3 of cortex	3	olfactory cortex	olf
dorsal endopiriform nucleus	DEn	olfactory cortex	olf
intermediate endopiriform nucleus	IEn	olfactory cortex	olf
posterolateral corFcal amygdaloid nucleus	PLCo	olfactory cortex	olf
posterolateral corFcal amygdaloid nucleus, layer 1	PLCo1	olfactory cortex	olf
posteromedial corFcal amygdaloid nucleus	PMCo	olfactory cortex	olf
piriform cortex	Pir	olfactory cortex	olf
piriform cortex, layer 1	Pir1	olfactory cortex	olf
piriform cortex, layer 1a	Pir1a	olfactory cortex	olf
ventral endopiriform nucleus	VEn	olfactory cortex	olf
dorsal peduncular cortex	DP	septum	septum
lateral septal nucleus, intermediate part	LSI	septum	septum
medial septal nucleus	MS	septum	septum
nucleus of the verFcal limb of the diagonal band	VDB	septum	septum
fimbria of the hippocampus	fi	septum	septum
anteromedial thalamic nucleus	AM	thalamus	thal
anteroventral thalamic nucleus, dorsomedial part	AVDM	thalamus	thal
anteroventral thalamic nucleus, ventrolateral part	AVVL	thalamus	thal
angular thalamic nucleus	AngT	thalamus	thal
centrolateral thalamic nucleus	CL	thalamus	thal
central medial thalamic nucleus	CM	thalamus	thal
interanteromedial thalamic nucleus	IAM	thalamus	thal
laterodorsal thalamic nucleus, ventrolateral part	LDVL	thalamus	thal
lateral posterior thalamic nucleus, mediocaudal part	LPMC	thalamus	thal
mediodorsal thalamic nucleus, central part	MDC	thalamus	thal
mediodorsal thalamic nucleus, lateral part	MDL	thalamus	thal
mediodorsal thalamic nucleus, medial part	MDM	thalamus	thal
paracentral thalamic nucleus	PC	thalamus	thal
posterior intralaminar thalamic nucleus	PIL	thalamus	thal
posterior thalamic nuclear group	Po	thalamus	thal
posterior thalamic nuclear group, triangular part	PoT	thalamus	thal
reFcular thalamic nucleus	Rt	thalamus	thal
subgeniculate nucleus	SubG	thalamus	thal
ventral anterior thalamic nucleus	VA	thalamus	thal
ventral geniculate nucleus, layer 1	VG1	thalamus	thal
ventrolateral thalamic nucleus	VL	thalamus	thal
ventromedial thalamic nucleus	VM	thalamus	thal
stria medullaris of the thalamus	sm	thalamus	thal

Wires were located in 95 anatomical structures (Paxinos & Watson).

Locations were grouped into 27 broader functional groups.

Intact

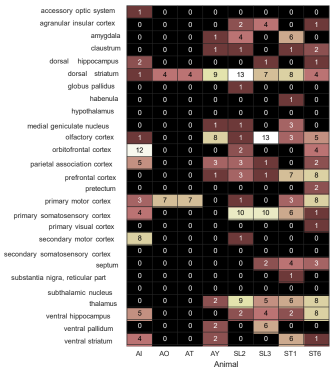

Lesioned

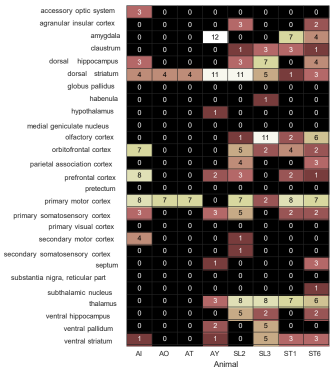

Supplementary Figure 1 – Overview of electrode locations.

Top left: Overview of the relative density of electrode tips located in respective structures in the eight individual rats. **Top right:** Summary of sub-structures grouped into 27 larger structures based on functional similarity. **Bottom:** Ensuing electrode density in the 27 larger structures divided per animal.

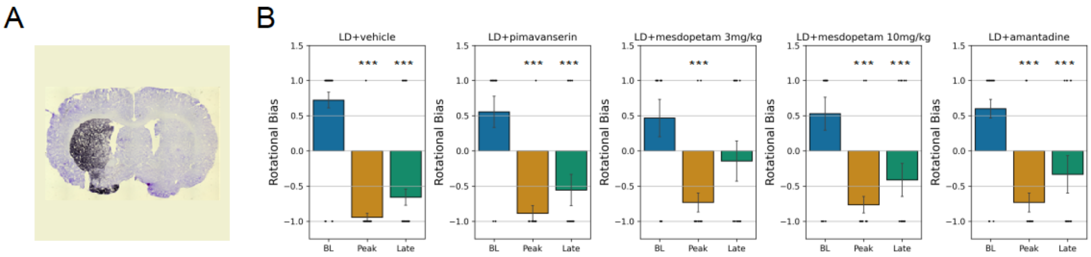

Supplementary Figure 2 – Assessment of 6-OHDA lesion severity.

A) Example brain section stained for tyrosine hydroxylase (TH) from a rat lesioned with 6-OHDA in the MFB of the right hemisphere, illustrating the extent of dopaminergic neuron loss. **B)** Rotational bias quantified as the net change in the cumulative angle shift over time, calculated by subtracting the initial value from the final value during each experimental period: Baseline (< -20 minutes), Peak (40–80 minutes), and Late (120–160 minutes). Positive and negative values of the net change indicate ipsilateral and contralateral rotational bias, respectively. Data are presented as Mean ± SEM, with individual data points representing recording sessions. Statistical significance was assessed using a GLME and Tukey-HSD post test. *** $p < 0.0001$.

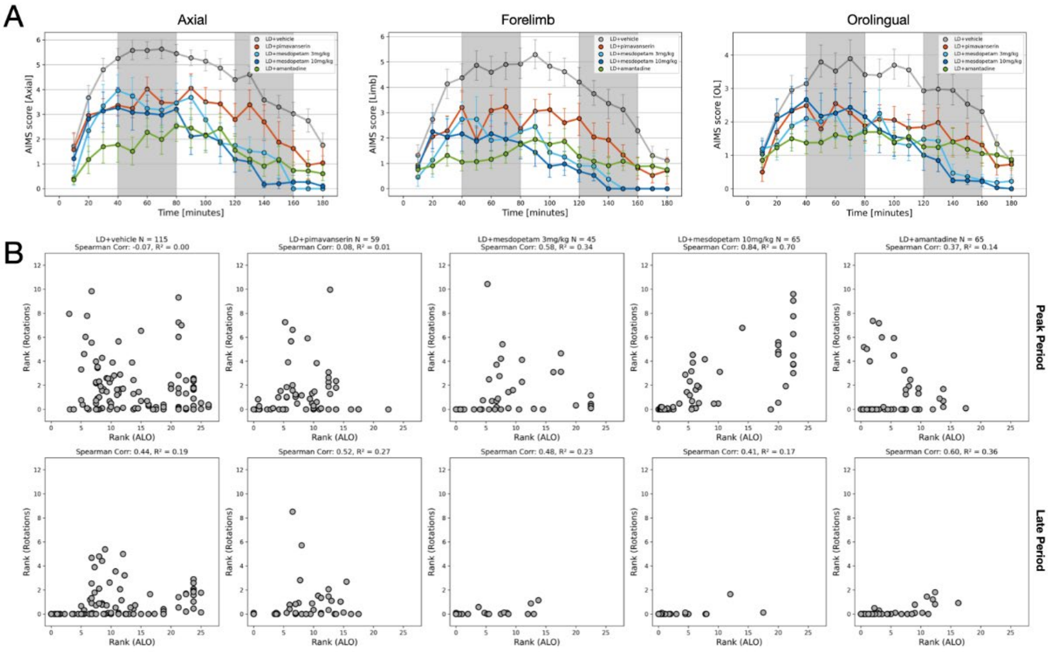

Supplementary Figure 3 - Quantification of abnormal involuntary movements involving different muscle groups.

A) Time course of Axial, Forelimb and Orolingual AIMs for each drug treatment after L-DOPA administration. Values represent AIMs scored during 1-min periods sampled every 10 min. For details on scoring procedures see Methods. **B)** Relationship between Rotational events and Global AIMs scores (ALO) for each treatment either during Peak (top row) or Late period (bottom row). Spearman correlation coefficients were calculated for the rank-transformed data to account for potential non-linear relationships between variables. Spearman correlation scores and R2 are shown over each graph. Circles represent datapoints in each time bin within the indicated period.
